# Steap4 Promotes Senile Osteoporosis via Fe^2+^‐ROS/C/EBPβ Feedback‐Driven Ferroptosis and Adipogenesis in Senescent BMSCs

**DOI:** 10.1002/advs.202509926

**Published:** 2025-11-07

**Authors:** Liangliang Wang, Guangrong Yin, Wenming Li, Maoyuan Li, Feng Lu, Chao Xu, Gongyin Zhao, Dechun Geng, Jiaxiang Bai, Yuji Wang

**Affiliations:** ^1^ Department of Orthopaedics the Second People's Hospital of Changzhou the Third Affiliated Hospital of Nanjing Medical University Changzhou Medical Center Nanjing Medical University Changzhou 213003 China; ^2^ Department of Orthopaedics The First Affiliated Hospital of Soochow University Suzhou 215006 China; ^3^ Department of Orthopedics Centre for Leading Medicine and Advanced Technologies of IHM The First Affiliated Hospital of USTC Division of Life Sciences and Medicine University of Science and Technology of China Hefei 230001 China; ^4^ Department of Orthopedic Surgery and Biochemistry and Molecular Biology Mayo Clinic Rochester MN 55902 USA

**Keywords:** BMSCs, ferroptosis, osteoporosis, senescence, Steap4

## Abstract

Senile osteoporosis (SOP) is a systemic bone disease characterized by increased susceptibility to fractures. In this study, it is found that senescent bone marrow mesenchymal stem cells (BMSCs) exhibit increased sensitivity to ferroptosis, a phenomenon associated with Steap4, a nicotinamide adenine dinucleotide phosphate hydrogen (NADPH)‐dependent metalloreductase that reduces Fe^3+^ to Fe^2+^. Therefore, it is aimed to innovatively elucidate how Steap4 affects ferroptosis in senescent BMSCs. These findings indicate that Steap4 promotes intracellular Fe^2+^ accumulation and elevates reactive oxygen species (ROS) levels, collectively driving the upregulation of CCAAT/enhancer binding protein beta (C/EBPβ) expression. Interestingly, a functional C/EBPβ binding site is identified within the Steap4 promoter region. Mechanistically, knockdown studies demonstrated that C/EBPβ depletion attenuated Steap4 expression, whereas C/EBPβ overexpression conversely upregulated Steap4 levels. These regulatory processes establish a self‐amplifying Steap4/Fe^2+^‐ROS/C/EBPβ positive feedback loop. Notably, a large number of adipocytes are also observed in the bone marrow of aged mice. Knockdown of Steap4 and C/EBPβ suppressed the differentiation of BMSCs into adipocytes. Knockdown of Steap4 or deferoxamine (DFO) treatment in animal experiments effectively relieves SOP. In conclusion, Steap4 accelerates the onset of ferroptosis in senescent BMSCs and promotes their differentiation to adipocytes through the Steap4/Fe^2+^‐ROS/C/EBPβ axis, ultimately impairing their osteogenic capacity.

## Graphical abstract

Senescent bone marrow mesenchymal stem cells (BMSCs) exhibit increased sensitivity to ferroptosis, a phenomenon associated with Steap4, a nicotinamide adenine dinucleotide phosphate hydrogen (NADPH)‐dependent metalloreductase that reduces Fe^3+^ to Fe^2+^. In addition, these regulatory processes establish a self‐amplifying Steap4/Fe^2+^‐ROS/C/EBPβ positive feedback loop. The finding provides a novel target for treating senile osteoporosis (SOP).

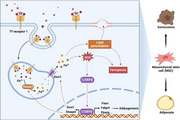

## 1 Introduction

Osteoporosis is a complicated bone disorders with grievous bone loss and skeletal microarchitectural deterioration. Osteoporosis is commonly divided into two categories, primary osteoporosis and secondary osteoporosis. The primary osteoporosis basically refers to three types, juvenile, postmenopausal, and senile osteoporosis (SOP), while the secondary osteoporosis is mostly caused by various diseases and drugs. SOP is a common bone metabolism disorder in the older population and is mainly characterized by decreased bone mass and abnormal bone microstructure, ultimately leading to increased bone fragility.^[^ ^1^ ^]^ Research focused specifically on senile osteoporosis carries profound practical significance. From a public health perspective, the global population is ageing at an unprecedented rate. Since the incidence of SOP and its associated fractures rises exponentially with age, this demographic shift will inevitably lead to a massive increase in the socioeconomic burden of the disease. Mechanisms involved in SOP are complicated and have not been fully elucidated. The abnormal function of bone marrow mesenchymal stem cells (BMSCs) plays an important role in the development of SOP, and the senescence of BMSCs is a key mechanism manifested by decreased proliferation ability and blocked differentiation.^[^ ^1^, ^2^ ^]^ These changes not only affect the normal renewal of BMSCs but also slow the speed of bone renewal. A growing number of studies are conducted to elucidate the mechanisms of senescence in BMSCs, and one of the hypotheses that has received much attention recently is that senescence in BMSCs may be associated closely with ferroptosis.^[^ ^3^ ^]^

Ferroptosis is a new type of programmed cell death characterized by iron‐dependent oxidative stress and injury, which ultimately causes excessive accumulation of lipid peroxides and triggers programmed cell death.^[^ ^4^ ^]^ These features generally resulted from the decline of glutathione synthesis and metabolism‐related functions in cells, which enhance the expression of reactive oxygen species (ROS). Iron is an essential trace element and an essential factor in redox reactions, which plays an important role in the electron transport chain and can participate in various enzymatic reactions as a coenzyme. Type I collagen is the main organic component of bone, and the transformation of precollagen to type I collagen requires the participation of iron ions.^[^ ^5^ ^]^ However, iron overload promotes collagen I degradation.^[^ ^6^ ^]^ In addition, excessive iron can lead to oxidative stress and cell damage. It has been reported that BMSCs in a high‐iron environment exhibit decreased survival, diminished proliferation and impaired differentiation, along with increased levels of apoptosis and oxidative stress.^[^ ^3^ ^]^ Zhang et al. measured iron levels in the femoral heads of 156 patients with femoral neck fractures and found that iron levels increased with age in patients with femoral neck fractures, with iron deposits on the edges and surfaces of trabeculae in older women, which were not observed in younger women.^[^ ^7^ ^]^ In addition, Kim et al. reported a negative correlation between bone mineral density of the femoral neck and serum ferritin in 1729 people undergoing physical examination;^[^ ^8^, ^9^ ^]^ subsequently, the team analyzed the strength of the femoral neck and serum ferritin in 693 women over the age of 45 and reported that high serum ferritin was associated with low femoral neck bone strength.^[^ ^9^ ^]^ Therefore, they suggested that iron accumulation can be an independent risk factor for reduced bone density. In conclusion, ferroptosis is closely related to BMSCs senescence, and further study of the mechanisms of ferroptosis and BMSCs senescence could help to elucidate the mechanisms of SOP.

Steap4 belongs to the six transmembrane epithelial antigen of prostate (STEAP) family, and it is involved in many biological processes, including the control of cell proliferation, apoptosis, oxidative stress and molecular transport in the endocytosis pathway.^[^ ^10^, ^11^, ^12^ ^]^ Steap family proteins are located within the Golgi apparatus and function as metal reductases that can use NAD(+) as an acceptor to reduce Fe^3+^ to Fe^2+^ and Cu^2+^ to Cu^1+^.^[^ ^10^, ^11^, ^12^ ^]^ In this study, RNA sequencing revealed that *Steap4* expression was significantly elevated in senescent BMSCs, which aroused our great interest.

BMSCs extracted from both humans and mice highly express Steap1 and Steap2, and inhibition of Steap1 expression reduces cell adhesion to culture dishes.^[^ ^13^ ^]^ In addition, the deletion of Steap4 in macrophages results in a limited ferrous iron supply, reduced mitochondrial ROS production, and impaired osteoclastogenesis.^[^ ^14^ ^]^ However, there are no studies on the effects of Steap4 on BMSCs, and the underlying mechanism remains unclear. In addition, the relationship between Steap4 and SOP is also not clear. Recent studies have shown that Steap4 contained in exosomes is translocated into the bacterial cytosol and induces Fe^2+^ elevation, ultimately causing ferroptosis.^[^ ^15^ ^]^ Furthermore, the Steap family also induces ferroptosis by reducing Cu^2+^ to Cu^1+^, which causes cellular lipid peroxidation.^[^ ^16^ ^]^

Therefore, we hypothesized that an abnormal increase in Steap4 in BMSCs may contribute to the pathogenesis of SOP by leading to uncontrolled intracellular redox homeostasis and thereby inhibiting osteogenic differentiation. Then, we found that Steap4 promoted Fe^2+^ accumulation and ROS generation. Moreover, Steap4 also stimulated CCAAT/enhancer binding protein beta (C/EBPβ or Cebpb) overexpression through the accumulation of ROS. Interestingly, we further identified a C/EBPβ binding site in the Steap4 promoter region, allowing C/EBPβ to activate the Steap4 promoter to increase Steap4 expression, forming a positive feedback loop: Steap4/Fe^2+^‐ROS/C/EBPβ, which in turn accelerates BMSCs oxidative stress and senescence. In summary, our findings suggest that Steap4 disrupts redox homeostasis in BMSCs and promotes BMSCs senescence in SOP, which provides a novel target for treating SOP.

## 2 Results

### 2.1 BMSCs from Ageing Mice are More Susceptible to Ferroptosis

**Figure 1:**
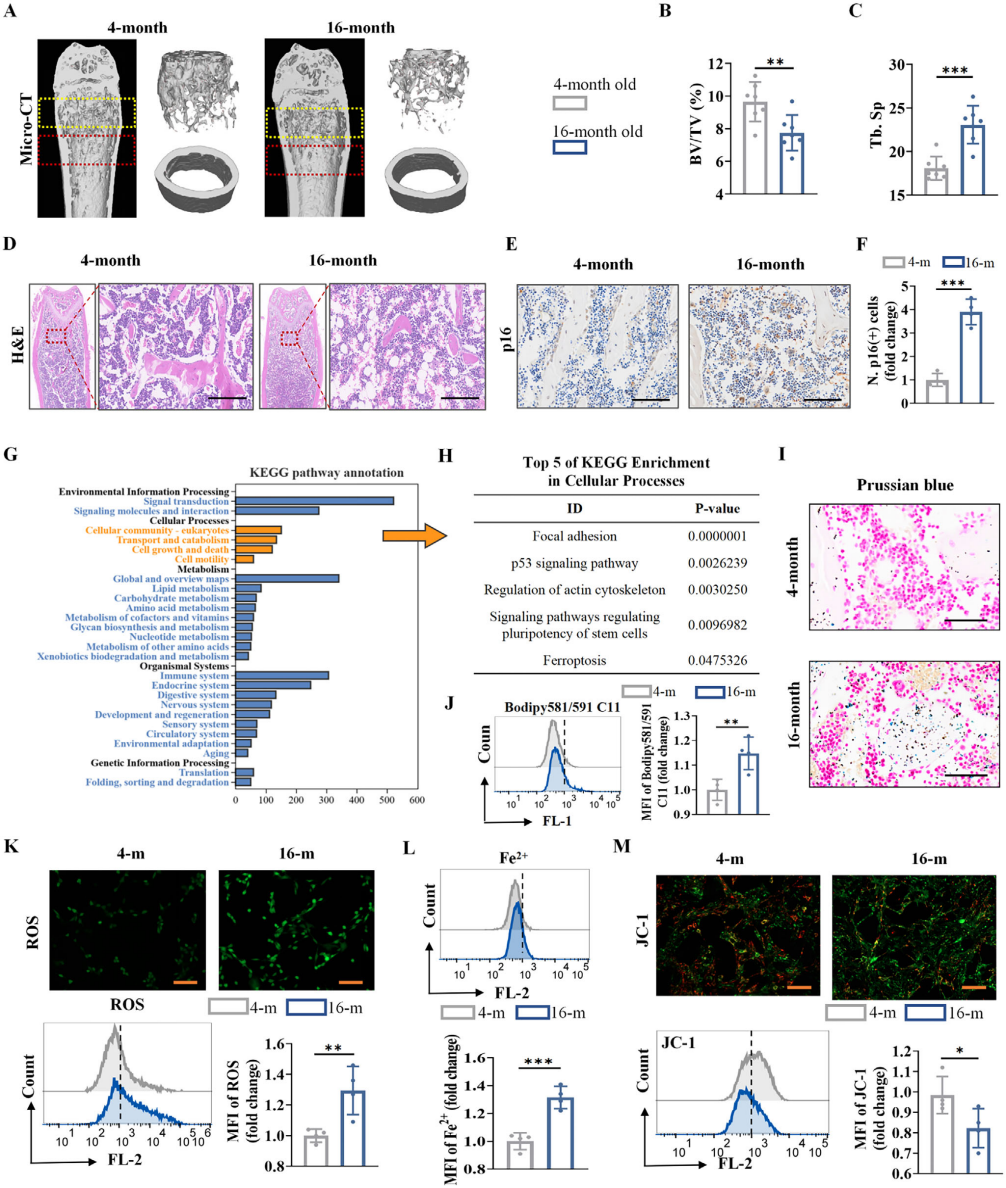
Ferroptosis of osteoblasts is involved in osteoporosis during ageing. A) Femoral micro‐CT scanning and 3D reconstruction. B) Bone volume/total volume (BV/TV) and C) trabecular separation (Tb.Sp.) were analyzed using femoral 3D reconstruction data (*n* = 7 per group). D) H&E staining (scale bar: 100 µm). E) Immunohistochemistry (IHC) staining for p16 (scale bar: 100 µm). F) Quantitative analysis of p16(+) cells (*n* = 4 per group). G,H) KEGG enrichment analysis. I) Prussian blue staining: the blue particles are iron‐containing particles (scale bar: 50 µm). J) BODIPY 581/591 C11 was used to detect lipid peroxides. K) DCFH‐DA was used to detect ROS, and images were observed using fluorescence microscopy and statistically quantified by flow cytometry (scale bar: 50 µm). L) Detection of intracellular Fe^2+^. M) JC‐1 was used to evaluate mitochondrial membrane potential, observed via fluorescence microscopy and quantified via flow cytometry (scale bar: 50 µm). (The data are presented as the means ± SDs. Statistical analysis was performed using one‐way ANOVA. ***p < 0.001, **p < 0.01, *p < 0.05, in vitro experiments *n* = 4.).

In this study, we first constructed an ageing animal model using 16‐month‐old mice and observed a reduction in bone tissue volume and trabeculae by micro‐computed tomography (CT) analysis (**Figure** **1**A–C). Consistent with the findings of a previous study, tartrate‐resistant acid phosphatase (TRAP) and alkaline phosphatase (ALP) staining in bone histological analyses revealed that the surface of bone trabeculae in aged mice was covered with increased numbers of TRAP‐positive osteoclasts and decreased numbers of ALP‐positive osteoblasts (Figure S1A,B, Supporting Information). Hematoxylin and eosin (H&E) staining showed that the bone trabeculae were sparse and more lipid‐like vacuoles were found in the bone marrow cavity of 16‐month‐old mice (Figure 1D). Immunohistochemical staining revealed a significant increase in p16(+) cells in the bone marrow of aged mice, indicating that the bone marrow exhibited senescent features (Figure 1E,F).

To further investigate what changes occurred in the BMSCs of aged mice, we performed RNA sequencing analysis on senescent BMSCs: BMSCs from 4‐month‐old mice were treated with osteogenic medium for 3 days, and BMSCs from 16‐month‐old mice were treated with osteogenic medium for 3 days. As shown in Figure S1C,D (Supporting Information), a total of 1244 downregulated and 3565 upregulated differentially expressed genes (DEGs) were identified. KEGG enrichment analysis revealed that functional changes in senescent BMSCs were mainly related to environmental information processing, cellular processes, metabolism, organismal systems and genetic information processing (Figure 1G). Then, we selected cellular processes from the KEGG analysis and found that ferroptosis was among the top 5 cellular processes (Figure 1H). Gene set enrichment analysis (GSEA) also revealed that bone development and osteoblast differentiation processes were negatively correlated with senescent BMSCs, whereas intracellular iron ion homeostasis and iron sulfur cluster assembly processes were more strongly enriched in senescent BMSCs (Figure S1E–H, Supporting Information). Besides, increased levels of iron ions were detected in the bone marrow of aged mice by Prussian blue staining (Figure 1I). Furthermore, we found that while serum iron levels were relatively lower in aged mice, levels in the bone marrow cavity were significantly higher (Figure S1I,J, Supporting Information), suggesting greater iron deposition in the bone marrow of aged mice. Therefore, we evaluated ferroptosis‐related changes in senescent cells. The results showed that senescent BMSCs from 16‐month‐old mice accumulated more lipid peroxides, ROS and Fe^2+^ than did BMSCs from 4‐month‐old mice (Figure 1J–L). In addition, the mitochondrial membrane potential was decreased in senescent BMSCs, suggesting mitochondrial insufficiency (Figure 1M).

**Figure 2:**
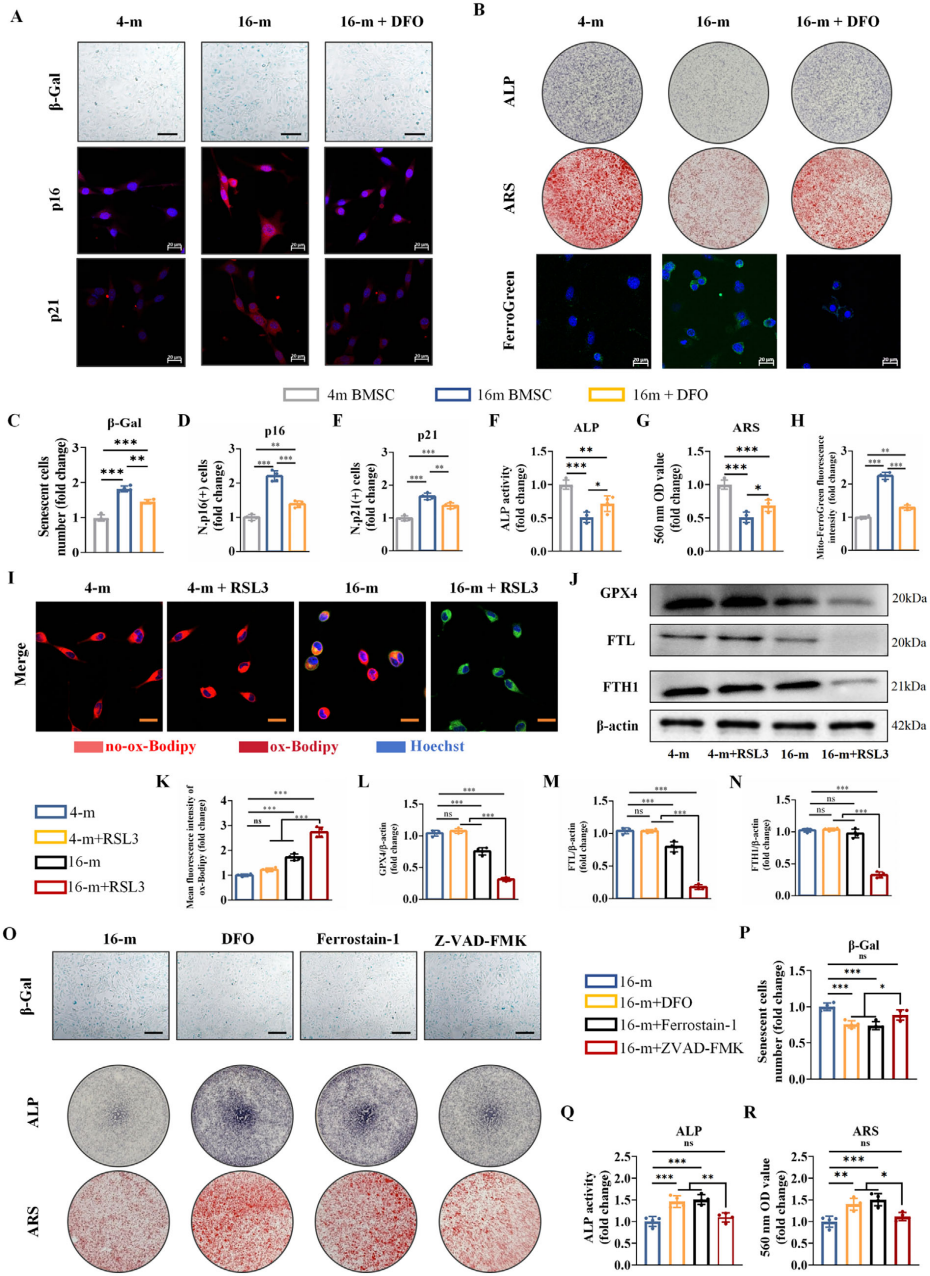
BMSCs from ageing mice are more susceptible to ferroptosis. A) Senescence β‐Gal staining (scale bar: 50 µm), immunofluorescence staining of p16 (red for p16 and blue for nuclei, scale bar: 20 µm) and p21 (red for p21 and blue for nuclei, scale bar: 20 µm). B) ALP staining, ARS staining and FerroGreen Assay (green for FerroGreen and blue for Hoechst, scale bar: 20 µm). C–E) Quantitative analysis of β‐Gal, p16, and p21. F–H) Quantitative analysis of ALP staining, ARS staining and FerroGreen Assay. I) Images of the BMSCs after coimmunostaining: red (no‐ox‐Bodipy for non‐lipid peroxidation), green (ox‐Bodipy for lipid peroxidation) and blue (hoechst, scale bar: 20 µm). J) GPX4, FTL, and FTH1 protein levels, detected by western blot analysis. Quantitative analysis of K) ox‐Bodipy, L) GPX4, M) FTL, and N) FTH1. O) Senescence β‐Gal staining, ALP staining and ARS staining (scale bar: 50 µm). Quantitative analysis of P) β‐Gal staining, Q) ALP staining, and R) ARS staining. (The data are presented as the means ± SDs. Statistical analysis was performed using one‐way ANOVA. ***p < 0.001, **p < 0.01, *p < 0.05, ns: not significant, *n* = 4.).

Subsequently, considering the role of osteoblast ferroptosis involved in osteoporosis during ageing, we further investigated the effects of intervention by ferroptosis modulators on the ageing and osteogenic differentiation of osteoblasts. Deferoxamine (DFO) is a ferric ion chelator and an inhibitor of ferroptosis, which was subsequently applied to BMSCs from 16‐month‐old mice. Senescence β‐galactosidase (β‐Gal) staining revealed that the proportion of senescent BMSCs was significantly greater in 16‐month‐old mice than in 4‐month‐old mice, but the percentage of senescent BMSCs was reduced by 20 µm DFO intervention (**Figure** **2**A,C). Meanwhile, based on the immunofluorescence assay, senescence markers of p16, p21, and p53 were higher in 16‐month‐old mice than in 4‐month‐old mice, but the percentage of senescent markers was decreased after DFO intervention (Figure 2A,D,E; Figure S2A,B, Supporting Information). The number of ALP‐positive cells was increased in senescent BMSCs after DFO treatment (Figure 2B,F). Meanwhile, Alizarin Red S (ARS) staining indicated that mineralization in senescent BMSCs was enhanced by DFO treatment (Figure 2B,G). Western blot analysis revealed that DFO treatment increased Runx2, Osterix, and osteocalcin (OCN) expression at the protein level in senescent BMSCs (Figure S2C–F, Supporting Information). All the results showed DFO alleviated the diminished osteogenesis of BMSCs in 16‐month‐old mice. Higher expression of ferrous ions in mitochondria was found in 16‐month‐old mice than in 4‐month‐old mice based on the FerroGreen Assay(Figure 2B,H). Likewise, DFO treament significantly reduced ferrous ions in ageing mice. RSL3 is an activator of ferroptosis and was also applied to BMSCs. More expression of lipid peroxidation was found in BMSCs from 16‐month‐old mice than that in 4‐month‐old mice. After the application of RSL3, lipid peroxidation in BMSCs from 16‐month‐old mice was highly increased (Figure 2I,K). Similarly, western blot analysis revealed the expression of GPX4, FTL and FTH1 was decreased significantly in BMSCs from 16‐month‐old mice by the treatment of RSL3 (Figure 2J,L–N). After treatment with the ferroptosis inhibitors DFO (20 µm) or ferrostatin‐1 (3 µm), the proportion of senescent BMSCs and the percentage of senescent markers were reduced, whereas treatment with the apoptosis inhibitor Z‐VAD‐FMK (50 µm) had no significant effect on senescence (Figure 2O,P; Figure S2G–I, Supporting Information). Similarly, the osteogenic capacity of BMSCs was restored by DFO or ferrostatin‐1 treatment, whereas treatment with Z‐VAD‐FMK had no significant effect on osteogenesis (Figure 2O,Q,R; Figure S2J–M, Supporting Information). Therefore, BMSCs from ageing mice are more susceptible to ferroptosis, and the inhibition of ferroptosis restores the osteogenic differentiation of senescent BMSCs.

### 2.2 Steap4 is Involved in Senescent BMSCs Ferroptosis

**Figure 3:**
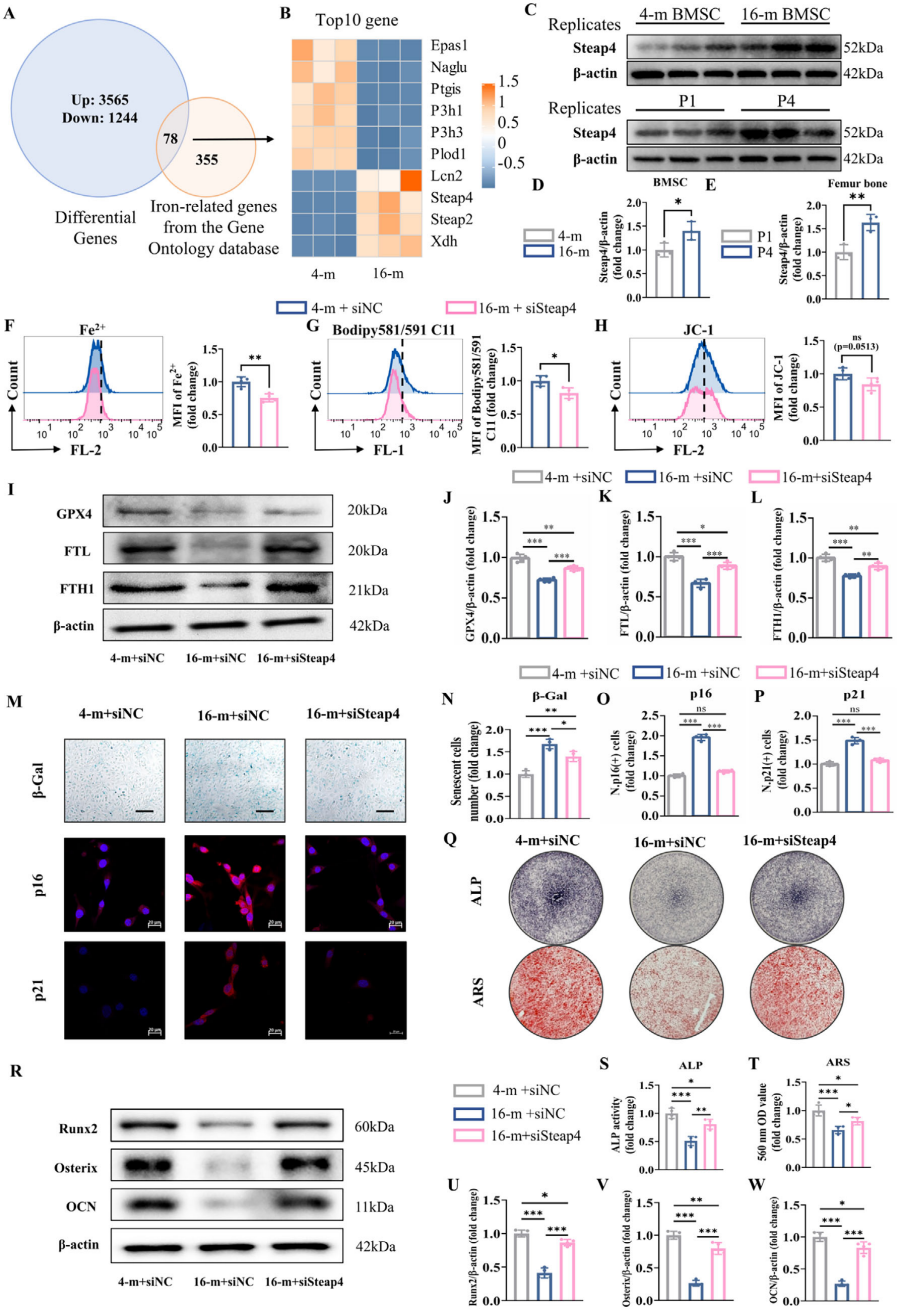
Steap4 is involved in senescent BMSCs ferroptosis. A) Venn analysis. B) The top 10 DEGs identified by Venn diagram analysis. C–E) Steap4 expression in BMSCs from 4‐month‐old and 16‐month‐old mice (*n* = 3 per group); and Steap4 expression was detected after extracting BMSCs from 4‐month‐old mice and culturing them into the first generation (P1) and fourth generation (P4). F) Detection of intracellular Fe^2+^. G) BODIPY 581/591 C11 was used to detect lipid peroxides. H) JC‐1 was used to evaluate the mitochondrial membrane potential. I) GPX4, FTL, and FTH1 protein levels, detected by western blot analysis. Quantitative analysis of J) GPX4, K) FTL, and L) FTH1. M) Senescence β‐Gal staining (scale bar: 50 µm), immunofluorescence staining of p16 (red for p16 and blue for nuclei, scale bar: 20 µm) and p21 (red for p21 and blue for nuclei, scale bar: 20 µm). Quantitative analysis of N) β‐Gal staining, O) p16, and P) p21. Q) ALP staining and ARS staining. R) Runx2, Osterix and OCN protein levels, detected by western blot analysis. Quantitative analysis of S) ALP staining, T) ARS staining, U) Runx2, V) Osterix, and W) OCN. (The data are presented as the means ± SDs. Statistical analysis was performed using one‐way ANOVA. ***p < 0.001, **p < 0.01, *p < 0.05, ns: not significant, *n* = 4.).

According to the results shown in Figures 1 and 2, we found that intracellular iron ion homeostasis plays an important role in BMSCs senescence and that the reduction of iron ions with DFO facilitated the osteogenic differentiation of BMSCs. Therefore, iron homeostasis‐related genes from the Gene Ontology database were used to intersect with DEGs from RNA‐seq analysis of BMSCs from 4‐month‐old and 16‐month‐old mice (**Figure** **3**A). A total of 78 DEGs were identified, among which the top 10 DEGs are presented in Figure 3B. KEGG analysis of the 78 DEGs revealed that ferroptosis was also one of the top 5 processes (Figure S3, Supporting Information). In addition, GSEA showed that the iron ion transmembrane transport process was more enriched in the senescent BMSCs group, and the Steap4 gene played a major role in this process (Figure S4, Supporting Information). Western blotting analysis further demonstrated that the expression of the Steap4 increased in BMSCs from 16‐month‐old mice, and the number of BMSCs that passed to the fourth generation also with enhanced steap4 expression (Figure 3C–E).

Therefore, we attempted to observe the changes in the status of senescent BMSCs by inhibiting Steap4 expression. We used siSteap4 siRNA to inhibit Steap4 expression. Two kinds of target sequences of siSteap4 were designed, and siSteap4(2) had a more significant inhibitory effect (Figure S5A,B, Supporting Information). Thus, siSteap4(2) was used in subsequent experiments. As shown in Figure 3F–H, the reduction in Steap4 attenuated the accumulation of Fe^2+^ in senescent BMSCs, alleviated lipid peroxidation and restored the mitochondrial membrane potential. In addition, higher expression of ferrous ions in mitochondria was found in 16‐m+siNC group based on the FerroGreen Assay(Figure S5C,D, Supporting Information), but knockdown of Steap4 significantly reduced ferrous ions in senescent BMSCs. Moreover, western blot analysis revealed the expression of GPX4, FTL, and FTH1 was increased significantly in senescent BMSCs by the treatment of siSteap4 (Figure 3I–L), which suggested that knockdown of Steap4 can inhibit ferroptosis in senescent BMSCs. Furthermore, β‐Gal staining showed that the proportion of senescent BMSCs decreased after siSteap4 treatment, and senescence markers of p16 and p21 were decreased in senescent BMSCs by the treatment of siSteap4 based on the immunofluorescence assay (Figure 3M–P). The number of ALP‐positive cells was increased in senescent BMSCs after siSteap4 treatment (Figure 3Q,S). Meanwhile, ARS staining indicated that mineralization in senescent BMSCs was enhanced by siSteap4 treatment (Figure 3Q,T). The results of real‐time quantitative polymerase chain reaction (RT‒qPCR) demonstrated the expression of marker genes (*Runx2*, *Osterix*, and *Ocn*) was increased in senescent BMSCs by the treatment of siSteap4 (Figure S5E–G, Supporting Information). Similarly, western blot analysis revealed that knockdown of Steap4 increased Runx2, Osterix, and OCN expression at the protein level (Figure 3R,U–W). All the results showed the osteogenic capacity of senescent BMSCs was restored by siSteap4 treatment. Therefore, Steap4 is involved in ferroptosis in senescent BMSCs. Knockdown of Steap4 can inhibit ferroptosis, alleviate senescence and enhance the osteogenic capacity in BMSCs.

### 2.3 Steap4 Promotes the Accumulation of Fe^2+^ in BMSCs In Vitro and Knockdown of Steap4 Effectively Relieves SOP In Vivo

**Figure 4:**
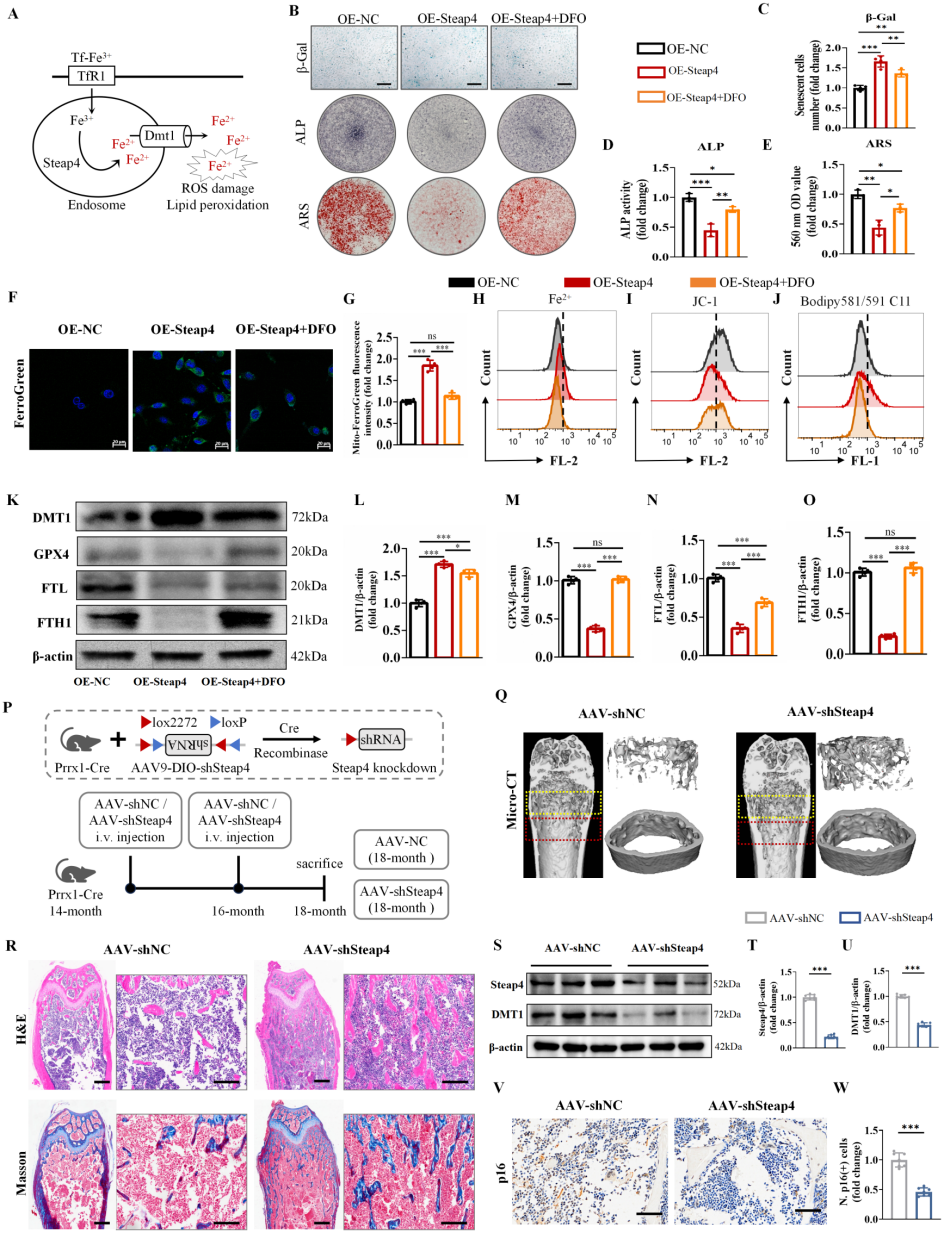
Steap4 promotes ferroptosis through the accumulation of Fe^2+^ in BMSCs. A) Graphical summary of the hypothesis. B) Senescence β‐Gal staining, ALP staining and ARS staining (scale bar: 50 µm). Quantitative analysis of C) β‐Gal staining, D) ALP staining and E) ARS staining. F) FerroGreen Assay (scale bar: 20 µm). G) Quantitative analysis of FerroGreen. H) Detection of intracellular Fe^2+^. I) JC‐1 was used to evaluate the mitochondrial membrane potential. J) BODIPY 581/591 C11 was used to detect lipid peroxides. K) DMT1, GPX4, FTL, and FTH1 protein levels, detected by western blot analysis. Quantitative analysis of L) DMT1, M) GPX4, N) FTL, and O) FTH1. P) Graphical abstract of the animal models. Q) Micro‐CT scanning and 3D reconstruction. R) H&E staining (scale bar: 200 µm of the overall picture and 500 µm of the partial enlarged picture) and Masson staining: blue‐stained tissue indicates newly formed bone, and red indicates mature bone tissue (scale bar: 200 µm of the overall picture and 500 µm of the partial enlarged picture). S) Steap4 and DMT1 protein levels, detected by western blot analysis. Quantitative analysis of T) Steap4 and U) DMT1. V) IHC staining of p16 (scale bar: 50 µm). W) Quantitative analysis of p16. (The data are presented as the means ± SDs. Statistical analysis was performed using one‐way ANOVA. ***p < 0.001, **p < 0.01, *p < 0.05, in vitro experiments *n* = 4, in vivo experiments *n* = 7.).

As shown in Figure S4 (Supporting Information), Steap4 played a major role in the iron ion transmembrane transport process in senescent BMSCs. In addition, previous studies have shown that Steap3 acts as a metalloreductase that reduces Fe^3+^ to Fe^2+^, which can be transported into the cytoplasm via DMT1.^[^ ^17^, ^18^ ^]^ Therefore, we hypothesized that steap4 promotes the reduction of Fe^3+^ to Fe^2+^ in senescent BMSCs and that DMT1 transports Fe^2+^ into the cytoplasm, thereby inducing ROS damage and lipid peroxidation (**Figure** **4**A).

To investigate the function of Steap4, a plasmid was constructed to transfect BMSCs overexpressing the Steap4 CDS region. When Steap4 was overexpressed in normal BMSCs (the OE‐Steap4 group), the cells exhibited a senescent phenotype with reduced osteogenic differentiation (Figure 4B–E; Figure S5J–M, Supporting Information). In contrast, treatment of Steap4‐overexpressing cells with DFO reduced the percentage of senescent cells and restored the osteogenic differentiation capacity (Figure 4B–E; Figure S5J–M, Supporting Information). In addition, higher expression of ferrous ions in mitochondria was found after the overexpression of Steap4 based on the FerroGreen Assay(Figure 4F,G), but the increase in ferrous ions was attenuated after DFO treatment. A Fe^2+^ detection assay showed that the overexpression of Steap4 promoted the accumulation of Fe^2+^ (Figure 4H). Moreover, the overexpression of Steap4 interfered with the mitochondrial membrane potential and exacerbated lipid peroxidation (Figure 4I,J). In contrast, treatment with DFO attenuated iron ion accumulation and restored the mitochondrial membrane potential and lipid peroxidation levels (Figure 4H–J). As expected, western blot analysis showed the overexpression of Steap4 significantly enhanced the expression of DMT1, whereas treatment with DFO inhibited the upward trend of DMT1 (Figure 4K,L). The expression of GPX4, FTL, and FTH1 was highly decreased in the OE‐Steap4 group. In contrast, treatment with DFO attenuated the percentage of ferroptosis‐related proteins (Figure 4K,M–O).

Considering the role of Steap4 in promoting ferroptosis in BMSCs, we applied adeno‐associated virus (AAV)‐DIO vectors to knock down Steap4 in BMSCs using Prrx1‐Cre mice as previously described (Figure 4P). Micro‐CT analysis revealed an increase in bone tissue volume and trabeculae in AAV‐shSteap4 group compared with AAV‐shNC group (Figure 4Q). Histopathological analysis of femur tissue by H&E and Masson staining further verified that knockdown of Steap4 in BMSCs by AAV‐shSteap4 effectively attenuated bone tissue loss compared with AAV‐shNC group (Figure 4R). Correspondingly, western blotting analysis also demonstrated that the expression of the Steap4 and DMT1 highly decreased in BMSCs from AAV‐shSteap4 group (Figure 4S–U). Meanwhile, IHC staining revealed that the expression of p16 was reduced in AAV‐shSteap4 group compared with AAV‐shNC group, suggesting that knockdown of Steap4 in BMSCs by AAV‐shSteap4 effectively slowed down the ageing process (Figure 4V,W). Therefore, Steap4 promotes the accumulation of Fe^2+^ in BMSCs, which causes oxidative damage, ferroptosis and decreased osteogenic capacity of BMSCs, whereas knockdown of Steap4 using Prrx1‐Cre mice effectively reduces bone loss and delays the process of senile osteoporosis.

### 2.4 The Accumulation of Fe^2+^ and ROS Stimulates C/EBPβ Transcriptional Activity to Promote Steap4 Expression

**Figure 5:**
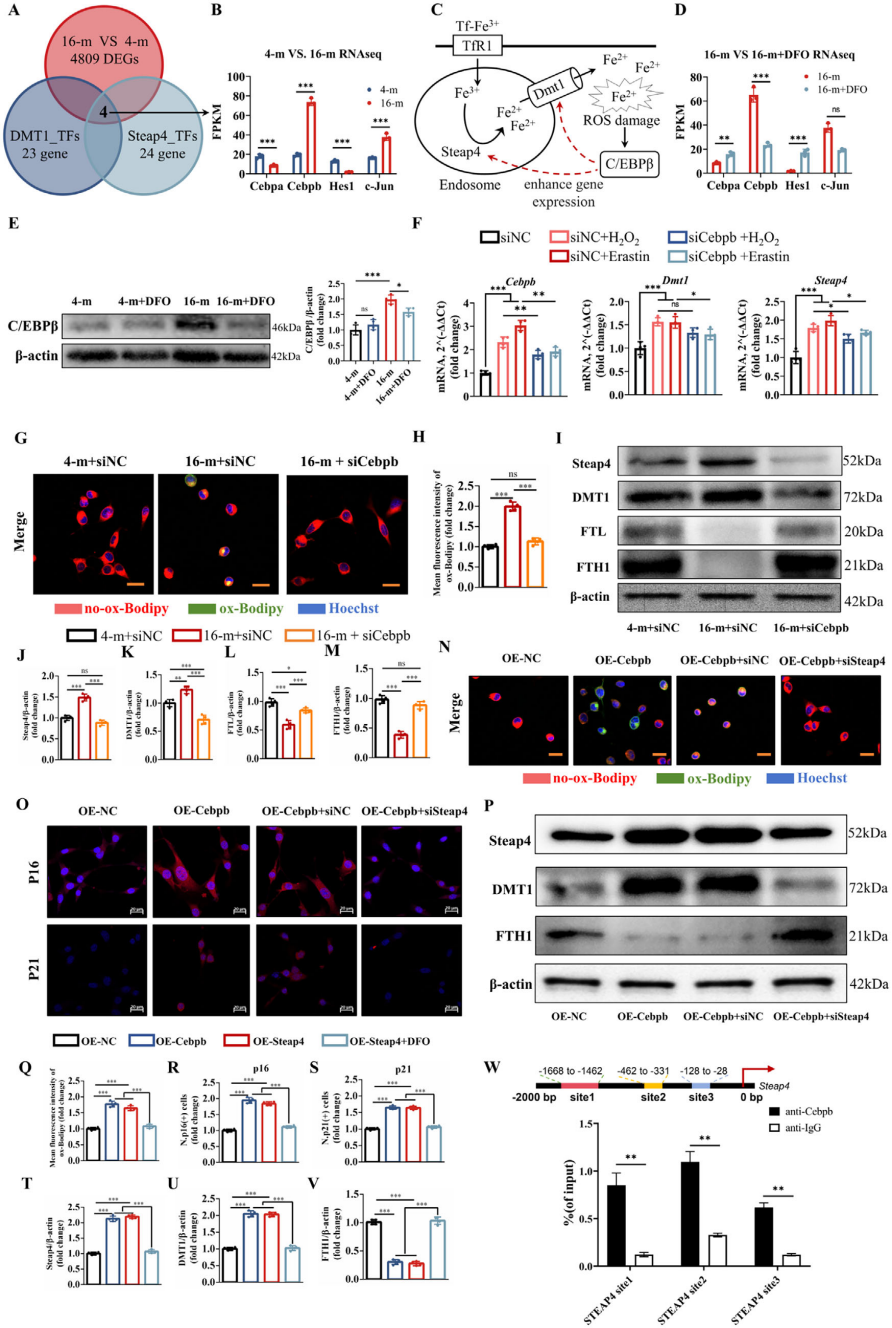
The Steap4/Fe^2+^‐ROS/C/EBPβ/Steap4 axis is involved in senescent BMSCs ferroptosis. A) Venn analysis of transcription factors and 16‐month versus 4‐month RNA‐seq results. B) FPKM of Cebpa, Cebpb, Hes1 and C‐Jun in 16‐month versus 4‐month RNA‐seq data. C) Graphical summary of the hypothesis. D) FPKM of Cebpa, Cebpb, Hes1, and C‐Jun in 16‐month VS 16‐month+DFO RNA‐seq results. E) Western blot analysis of C/EBPβ. F) RT‒qPCR of *Cebpb*, *Steap4*, and *Dmt1*. G) Images of the BMSCs after coimmunostaining: red (no‐ox‐Bodipy for non‐lipid peroxidation), green (ox‐Bodipy for lipid peroxidation) and blue (hoechst, scale bar: 20 µm). H) Quantitative analysis of ox‐Bodipy. I) Steap4, DMT1, FTL, and FTH1 protein levels, detected by western blot analysis. Quantitative analysis of J) Steap4, K) DMT1, L) FTL, and M) FTH1. N) Images of the BMSCs after coimmunostaining: red (no‐ox‐Bodipy for non‐lipid peroxidation), green (ox‐Bodipy for lipid peroxidation) and blue (hoechst, scale bar: 20 µm). O) Immunofluorescence staining of p16 (red for p16 and blue for nuclei, scale bar: 20 µm) and p21 (red for p21 and blue for nuclei, scale bar: 20 µm). P) Steap4, DMT1 and FTH1 protein levels, detected by western blot analysis. Quantitative analysis of Q) ox‐Bodipy, R) p16, S) p21, T) Steap4, U) DMT1, and V) FTH1. W) ChIP‐qPCR: Schematic diagram of three ChIP‐qPCR sites in the promoter region of the Steap4 gene, and the ChIP‐qPCR results. (The data are presented as the means ± SDs. Statistical analysis was performed using one‐way ANOVA. ***p < 0.001, **p < 0.01, *p < 0.05, *n* = 4.).

In addition, to further study the senescent cellular status of the increased Fe^2+^ accumulation by Steap4 as previously mentioned, we performed RNA‐seq to detect differences between senescent BMSCs and DFO‐treated senescent BMSCs (Figure S6A,B, Supporting Information). GSEA showed that ferroptosis process was more strongly enriched in the senescent BMSCs group, and bone mineralization was more strongly enriched in the DFO‐treated group (Figure S6C,D, Supporting Information). Interestingly, we found that the expression of both Steap4 and DMT1 was reduced in DFO‐treated BMSCs, although there was no significant difference in the reduction of DMT1 according to the RNA‐seq results (Figure S6B, Supporting Information). Therefore, we speculated that steap4 promotes Fe^2+^ accumulation in senescent BMSCs and that Fe^2+^ and ROS stimulate transcription factors to increase Steap4 expression, forming a positive feedback loop. In this study, the transcription factor‐binding site prediction website (PROMO) was used to detect the transcription factors of Steap4 and DMT1 in promoter regions, and the results are shown in Figure S7 (Supporting Information). Moreover, the intersection between the DEGs identified in senescent BMSCs and the transcription factors of Steap4 and DMT1 suggested that Cebpa, Cebpb, Hes1, and C‐Jun may be potential targets (**Figure** **5**A,B). The FPKM values of Cebpa, Cebpb, Hes1, and C‐Jun in the RNA‐seq results showed that the most significant changes were observed in Cebpb (Figure 5B). Therefore, we hypothesized that C/EBPβ is involved in a positive feedback loop that promotes ferroptosis in senescent BMSCs (Figure 5C).

In addition, the RNA‐seq results showed that *Cebpb* was also downregulated in DFO‐treated senescent BMSCs (Figure 5D). Western blot analysis further confirmed that C/EBPβ decreased after DFO treatment in senescent BMSCs (Figure 5E). When BMSCs were treated with H_2_O_2_ or Erastin to induce oxidative damage, *Cebpb*, *Steap4*, and *Dmt1* were upregulated (Figure 5F). However, the expression of *Steap4* and *Dmt1* was alleviated in siCebpb‐treated BMSCs during H_2_O_2_ or Erastin treatment (Figure 5F). In order to conduct an in‐depth study of the relationship between Steap4 and C/EBPβ, lipid peroxidation was detected in siCebpb‐treated BMSCs. The results showed that lipid peroxidation in BMSCs from 16‐month‐old mice was highly reduced after the application of siCebpb (Figure 5G,H). Moreover, western blot analysis showed the treatment with siCebpb significantly reduced the expression of Steap4 and DMT1, whereas the expression of FTL, FTH1, and GPX4 was highly increased in the 16‐m+siCebpb group (Figure 5I–M; Figure S8A,B, Supporting Information). Overall, these results are in line with our speculation that C/EBPβ is involved in a positive feedback loop that promotes ferroptosis in senescent BMSCs.

In order to better verify our hypothesis, Cebpb was overexpressed in senescent BMSCs (the OE‐Cebpb group). Lipid peroxidation in BMSCs from 16‐month‐old mice was highly enhanced after the application of OE‐Cebpb (Figure S9A,C, Supporting Information). Western blot analysis showed the treatment with OE‐Cebpb significantly enhanced the expression of Steap4, DMT1 and TFRC (Figure 5P,T,U; Figure S9B,D,E, Supporting Information). Furthermore, more expression of ferrous ions in mitochondria was found after the overexpression of Cebpb based on the FerroGreen Assay (Figure S9F,G, Supporting Information). Subsequently, siSteap4 was used to figure out the deep‐seated connection between Steap4 and C/EBPβ. Lipid peroxidation in OE‐Cebpb‐treated BMSCs from 16‐month‐old mice was significantly reduced after the application of siSteap4 (Figure 5N,Q). Senescence markers of p16 and p21 were highly increased in senescent BMSCs by the treatment of OE‐Cebpb based on the immunofluorescence assay (Figure 5O,R,S), whereas the treatment with siSteap4 reduced the upward trend of p16 and p21 induced by OE‐Cebpb. In addition, western blot analysis showed the treatment with siSteap4 not only reduced the upward trend of Steap4 and DMT1 induced by OE‐Cebpb, but also enhanced the expression of FTH1, which was previously decreased by the treatment with OE‐Cebpb (Figure 5P,T–V). Next, we tested whether C/EBPβ could bind to the promoters of Steap4. To determine the binding of Cebpb to the cis‐regulatory elements of the mouse Steap4 gene, we selected three sites within the Steap4 promoter region (from 0 to −2000 bp upstream of the Steap4 gene) for chromatin immunoprecipitation assay (ChIP)‐qPCR analysis (Figure 5W). ChIP‐qPCR results revealed that the Cebpa group exhibited significantly stronger binding to the Steap4 promoter region compared to the IgG control group. Among the three sites, Site2 showed the strongest binding.

Therefore, Steap4 promotes Fe^2+^ accumulation in senescent BMSCs, and then, Fe^2+^ and ROS stimulate C/EBPβ to increase steap4 expression, forming a positive feedback loop to promote cellular senescence.

### 2.5 The Steap4/C/EBPβ Axis Promotes the Differentiation of Senescent BMSCs into Adipocytes

**Figure 6:**
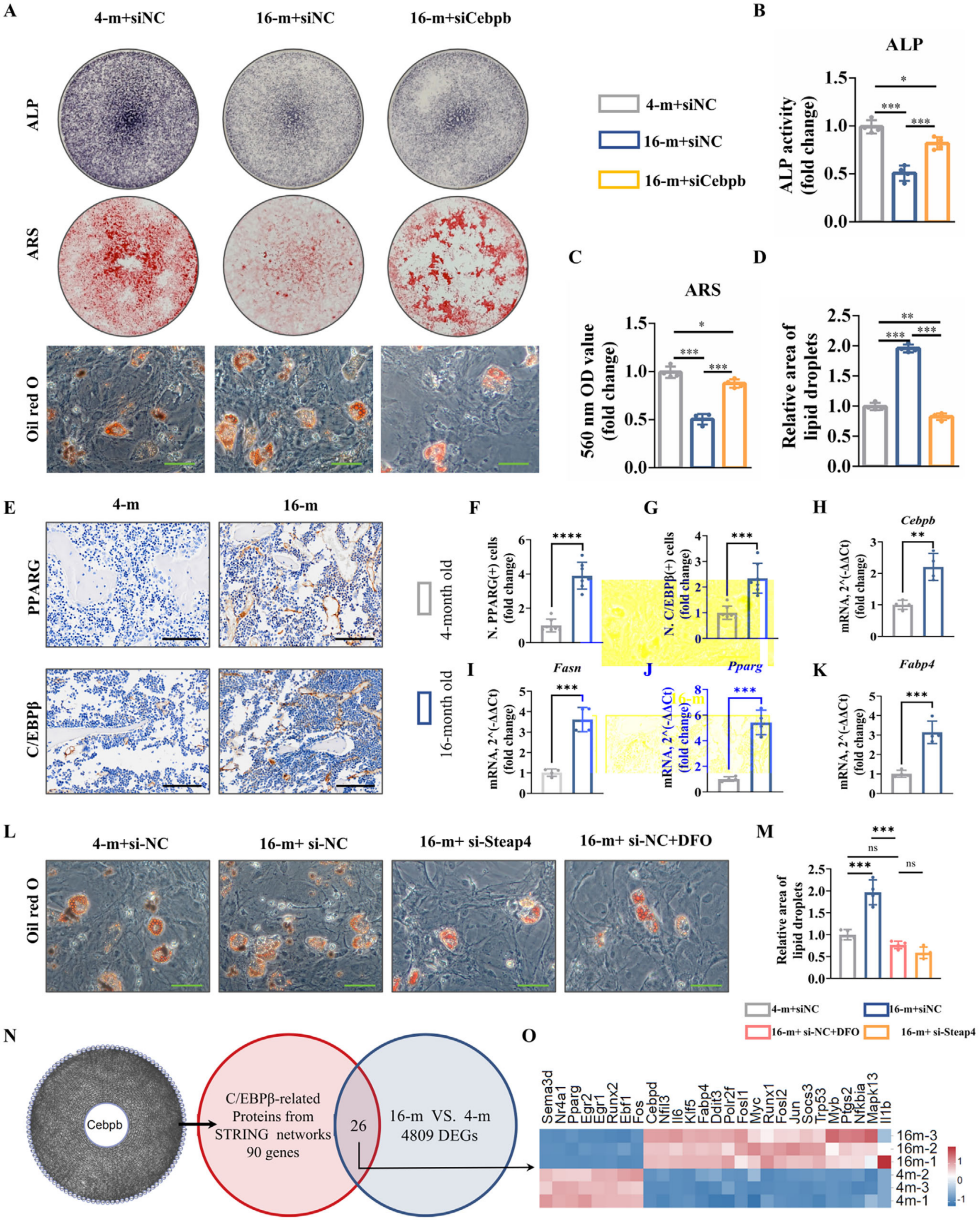
Steap4 is involved in the adipogenesis of senescent BMSCs. A) ALP staining, ARS staining and Oil red O staining (scale bar: 50 µm). Quantitative analysis of B) ALP staining, C) ARS staining, and D) Oil red O staining. E) IHC staining for PPARG and C/EBPβ (scale bar: 100 µm). Quantitative analysis of F) PPARG and G) C/EBPβ. RT‒qPCR of H) Cebpb, I) Fatty acid synthase (Fasn), J) Pparg, and K) Fatty acid binding protein 4 (Fabp4). L) Oil red O staining (scale bar: 50 µm). M) Quantitative analysis of Oil red O staining. N) Venn analysis of C/EBPβ‐related proteins from STRING networks and 16‐month VS 4‐month RNA‐seq results. O) Heatmap of the Venn analysis results. (The data are presented as the means ± SDs. Statistical analysis was performed using one‐way ANOVA. ***p < 0.001, **p < 0.01, *p < 0.05, in vitro experiments *n* = 4, in vivo experiments *n* = 7.).

BMSCs are involved in the remodeling and regeneration of bone tissue, whereas senescent BMSCs undergo increased transformation to bone marrow adipocytes and impaired differentiation to osteoblasts.^[^ ^19^ ^]^ In this study, ALP staining and ARS staining also showed that the osteogenic function of senescent BMSCs was improved after inhibition of C/EBPβ expression using siCebpb (**Figure** **6**A–C). Western blot analysis also revealed that inhibition of C/EBPβ expression by siCebpb increased Runx2, Osterix and OCN expression at the protein level in senescent BMSCs (Figure S8A,C–E, Supporting Information). In addition, a number of studies have shown that C/EBPβ plays an important role in promoting differentiation to adipocytes and regulating cellular metabolism.^[^ ^20^, ^21^ ^]^ Oil red O staining revealed that senescent BMSCs were more likely to differentiate into adipocytes, whereas the adipogenesis was inhibited by siCebpb treatment (Figure 6A,D). Moreover, normal adipose tissue also expresses Steap4, which plays an important role in the maintenance of proliferation, apoptosis and insulin sensitivity.^[^ ^22^, ^23^ ^]^ Therefore, we hypothesized that Steap4/C/EBPβ not only promotes oxidative stress in BMSCs but also promotes differentiation toward adipocytes.

IHC staining showed that the expression of C/EBPβ and peroxisome proliferator activated receptor gamma (PPARG) was both increased in the bone marrow of aged mice (Figure 6E–G). RT‒qPCR results also indicated that adipogenesis‐related genes, including *Cebpb*, *Fasn*, *Fabp4*, and *Pparg*, were upregulated in senescent BMSCs from aged mice (Figure 6H–K). Furthermore, Oil Red O staining showed that senescent BMSCs were more likely to differentiate into adipocytes, whereas inhibition of Steap4 in BMSCs or DFO treatment impaired their ability to differentiate into adipocytes (Figure 6L,M).

Finally, to gain insight into the transcriptional mechanisms driving senescent BMSCs to be more susceptible to adipogenesis and senescence, we performed Venn analysis between C/EBPβ‐related proteins from STRING networks and DEGs from 16‐month/4‐month BMSC RNA‐seq (Figure 6N). The intersecting genes are shown in Figure 6O. We observed that the expression of aging‐related genes, including IL6, IL1b, and JUN, was upregulated in BMSCs from 16‐month‐old mice. Consistent with this senescent profile, Cebpb was also elevated in aged mice, suggesting that it may promote a senescent phenotype by regulating IL6, IL1b, and JUN expression. Notably, Cebpd, a transcription factor belonging to the same bZIP family as Cebpb and sharing high phylogenetic and functional similarity, was also upregulated in aged mice. Since Cebpb and Cebpd are known to cooperate with other transcription factors and broaden their regulatory targets, the increased Cebpd expression implies it may assist Cebpb in promoting adipogenic differentiation. In line with this, fatty acid‐binding protein 4 (Fabp4) expression was also elevated, further supporting a predisposition of aged BMSCs toward adipocyte differentiation. This finding aligns with reports that Cebpb promotes Fabp4 transcription. Moreover, Fabp4 not only participates in fatty acid storage and catabolism but also acts as a key mediator in inflammatory responses. Collectively, these results suggest that the Steap4/C/EBPβ signaling axis promotes adipogenic differentiation in senescent BMSCs. Future studies should investigate how Steap4/C/EBPβ influences aging and inflammation, particularly its role in driving adipocytes to adopt a senescence‐associated secretory phenotype (SASP).

### 2.6 The Steap4/C/EBPβ Axis is Involved in Osteoporosis by Promoting Ferroptosis and Adipogenesis in BMSCs

**Figure 7:**
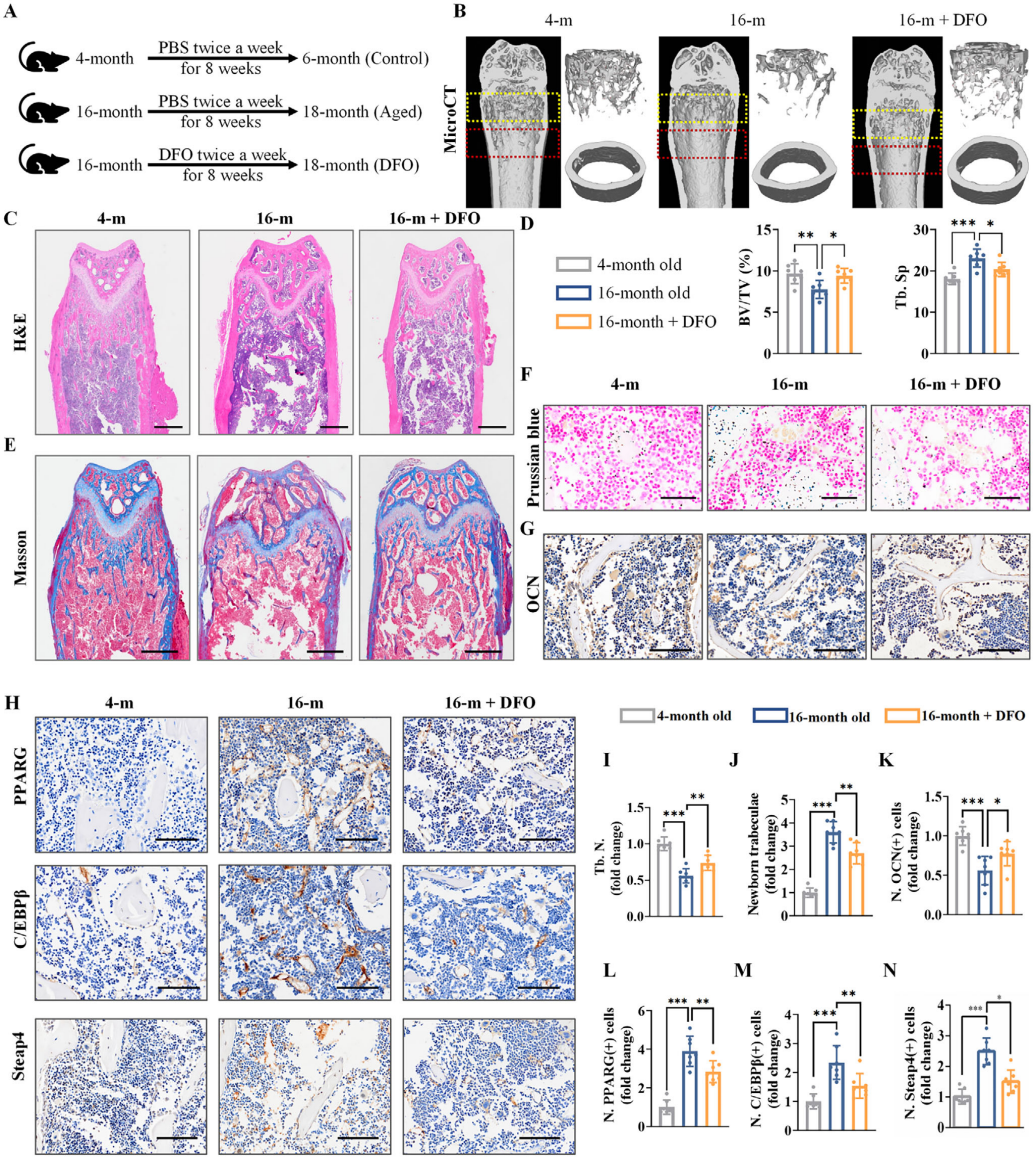
DFO effectively relieves SOP. A) Graphical abstract of the animal models. B) Micro‐CT scanning and 3D reconstruction. C) H&E staining (scale bar: 500 µm). D) Bone volume/total volume (BV/TV) and trabecular separation (Tb.Sp.) were analyzed using femoral 3D reconstruction data. E) Masson staining: blue‐stained tissue indicates newly formed bone, and red indicates mature bone tissue (scale bar: 500 µm). F) Prussian blue staining: the blue particles are iron‐containing particles (scale bar: 100 µm). G,H) IHC staining for OCN, PPARG, C/EBPβ and Steap4 (scale bar: 100 µm). I) Trabecular number (Tb.N.). J) Masson staining analysis of newborn trabeculae. K–N) Quantitative analysis of OCN(+), PPARG(+), C/EBPβ(+) and Steap4(+) cells. (The data are presented as the means ± SDs. Statistical analysis was performed using one‐way ANOVA. ***p < 0.001, **p < 0.01, *p < 0.05, *n* = 7.).

In the present study, we raised the mice to 16 months old and successfully constructed a mouse model of SOP that exhibited a reduction in femoral trabeculae (Figure 1A). As shown in **Figure** **7**A and 4‐month‐old and 16‐month‐old mice were performed with different treatment. Micro‐CT analysis revealed a reduction in bone tissue volume and trabeculae in 16‐month‐old mice compared with 4‐month‐old mice (Figure 7B,D). When 16‐month‐old mice were treated with DFO to reduce Steap4‐induced Fe^2+^ accumulation, the bone tissue volume and trabeculae increased (Figure 7B,D). Histopathological analysis of femur tissue by H&E and Masson staining further verified that DFO treatment attenuated bone tissue loss in aged mice (Figure 7C,E,I,J).

Prussian blue staining showed increases in iron ions in the bone marrow of aged mice (Figures 1I and 7F), and the accumulation of iron decreased after DFO treatment (Figure 7F). In addition, the expression of OCN decreased in aged mice and was restored after DFO treatment (Figure 7G,K). Meanwhile, IHC staining revealed that the expression of PPARG, C/EBPβ and Steap4 was elevated in the bone marrow of aged mice compared with 4‐month‐old mice, and 16‐month‐old mice treated with DFO exhibited decreased bone marrow PPARG, C/EBPβ and Steap4 expression, suggesting that the Steap4/Fe^2+^‐ROS/C/EBPβ positive feedback loop was inhibited (Figure 7H,L–N).

**Figure 8:**
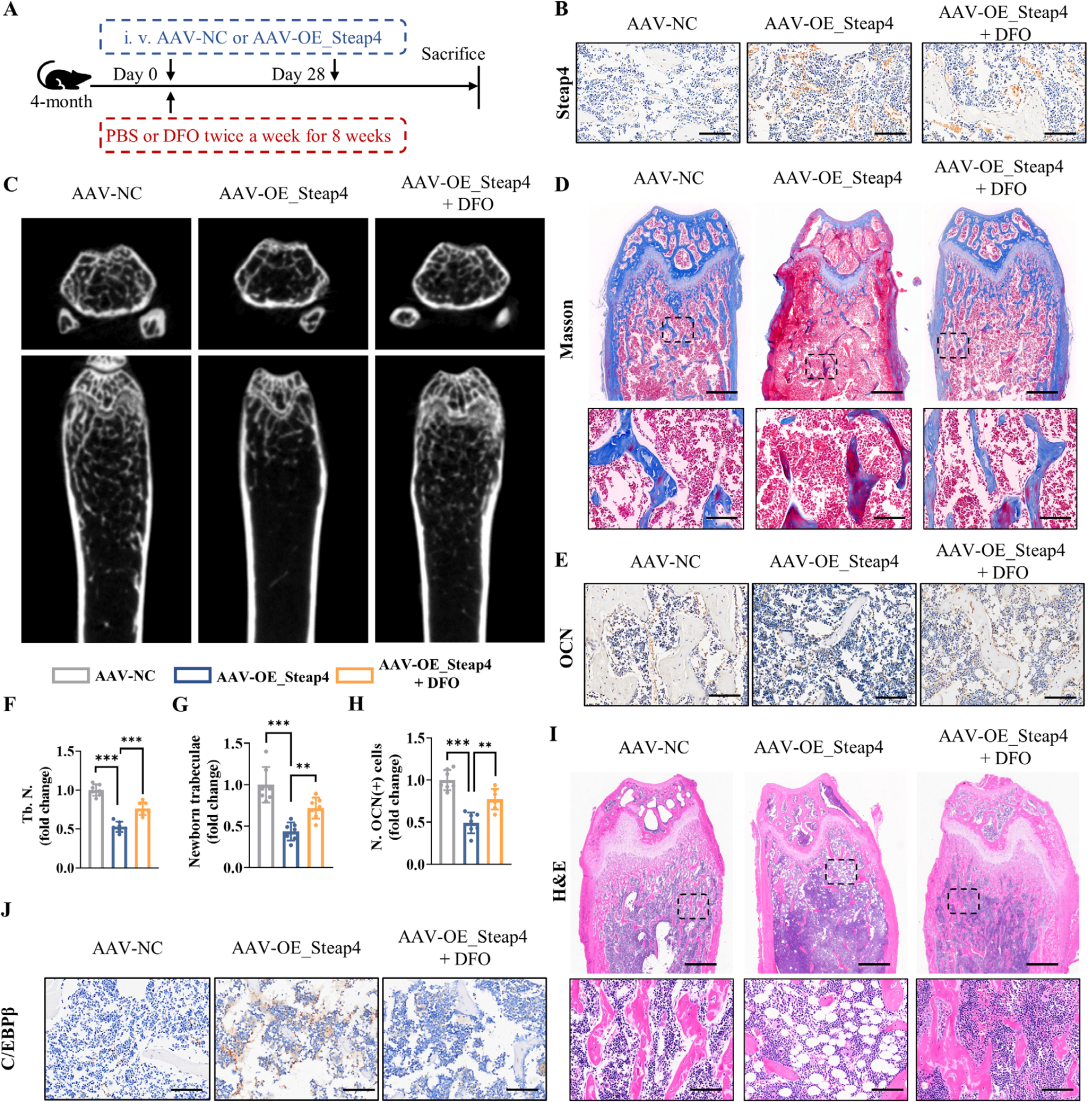
DFO effectively rescue Steap4‐induced insufficient osteogenic function. A) Graphical abstract of the animal models. B) IHC staining for Steap4 (scale bar: 100 µm). C) Micro‐CT scanning and reconstruction. D) Masson staining (scale bar: 500 µm and 100 µm). E) IHC staining for OCN (scale bar: 100 µm). F) Quantitative analysis of Micro‐CT scanning. G) Quantitative analysis of Masson staining. H) Quantitative analysis of OCN. (I) H&E staining (scale bar: 500 and 100 µm). J) IHC staining for C/EBPβ (scale bar: 100 µm). (The data are presented as the means ± SDs. Statistical analysis was performed using one‐way ANOVA. ***p < 0.001, **p < 0.01, *p < 0.05, *n* = 7.).

Furthermore, to validate whether DFO could rescue the Steap4‐mediated inhibition of bone formation in vivo, we overexpressed Steap4 (AAV‐OE_Steap4) in 4‐month‐old mice (**Figure** **8**A,B; Figure S10A, Supporting Information). Correspondingly, western blotting analysis also confirmed that the expression of the Steap4 highly increased in BMSCs from AAV‐OE_Steap4 group (Figure S10B,C, Supporting Information). Micro‐CT analysis showed that Steap4 overexpression reduced trabecular bone number compared to AAV‐NC mice, while DFO treatment reversed the trabecular bone number (Figure 8C,F). Histological analysis of osteogenic function via Masson and OCN staining further confirmed that Steap4 overexpression impaired bone formation, which was ameliorated by DFO treatment (Figure 8D,E,G,H). Additionally, H&E staining revealed a significant accumulation of lipid‐like vacuoles in the bone marrow upon Steap4 overexpression, and this phenotype was mitigated by DFO therapy (Figure 8I). Finally, DFO treatment counteracted the Steap4‐induced upregulation of C/EBPβ, further confirming the inhibitory effect of DFO treatment on Steap4/C/EBPβ (Figure 8J; Figure S10D, Supporting Information).

In conclusion, the Steap4/C/EBPβ axis is involved in osteoporosis by promoting ferroptosis and adipogenesis in BMSCs, whereas DFO treatment effectively inhibited the Steap4/C/EBPβ axis and osteoporosis.

## 3 Discussion

Osteoporosis is a systemic disease associated with abnormal bone metabolism characterized by reduced bone mineral density, impaired bone microstructure and increased bone fragility.^[^ ^1^ ^]^ The number and proportion of older persons is growing rapidly in most countries and zones, and the increase is thought to accelerate in the next several decades. As a result, the SOP has become a worldwide public health concern as it is an age‐related disorder, leading to low bone mass and deteriorated bone microstructure. More importantly, osteoporotic fractures are very dangerous, with high rates of disability and mortality, and early prevention can reduce the incidence of osteoporosis and its associated fractures.^[^ ^1^ ^]^ Bone is a dynamic tissue that requires constant remodeling and reconstruction of bone tissue to maintain function. It is confirmed that BMSCs can differentiate into osteoblasts, and changes in the activity and features of BMSCs are the main causes of SOP.^[^ ^24^, ^25^ ^]^ Cellular senescence is the main pathological mechanism leading to a variety of aging‐related diseases and bone loss as well. Therefore, it is evident that targeting cellular senescence and modulation of bone regeneration can be a promising approach to treat SOP.

Iron ions play a crucial role in maintaining redox balance, promoting various biochemical reactions and regulating enzyme activities to maintain normal biological activities in the human body. However, Zhang et al. reported that iron levels in patients with femoral neck fractures increased with age and that iron was deposited on the edges and surfaces of the trabeculae in older women but not in younger women.^[^ ^7^ ^]^ In this study, we observed increased levels of iron ions in the bone marrow of aged mice. In addition, DFO treatment of senescent BMSCs reduces senescence‐related β‐Gal staining and aging‐related marker proteins, and promotes osteogenic activity during the osteogenic induction of senescent BMSCs. Moreover, excess iron‐induced bone loss has been reported to play an important role in osteoporosis by inducing ferroptosis in senescent BMSCs, which is consistent with our subsequent findings.^[^ ^26^ ^]^ Next, we confirmed that the accumulation of iron ions in senescent BMSCs leads to ferroptosis through several experiments, including the detection of cellular ROS, lipid peroxidation, iron ions, changes in the mitochondrial membrane potential and ferroptosis‐related proteins. Meanwhile, we successfully mitigated the onset of ferroptosis in senescent BMSCs using DFO treatment, offering a potential therapeutic strategy for the treatment of SOP.

To further investigate how iron ions accumulate in senescent BMSCs, we performed RNA‐seq to determine which genes exhibited changes at the transcriptional level. Then, we observed that the iron ion transmembrane transport process was more enriched in the senescent BMSCs group, and the Steap4 gene played a major role in this process. The STEAP family are belong to metalloreductase and is associated with copper and iron homeostasis, is essential for maintaining cellular activity.^[^ ^16^, ^27^ ^]^ However, iron or copper overloading by Stesp4 reportedly leads to increased oxidative stress and ultimately induces lipid peroxidation and ferroptosis.^[^ ^16^, ^27^ ^]^ Therefore, we attempted to observe the changes in the status of senescent BMSCs by inhibiting Steap4 expression. Lipid peroxidation and iron metabolism were attenuated when we reduced Steap4 in senescent BMSCs. Moreover, β‐Gal staining and reduction of aging‐related marker proteins showed that the proportion of senescent BMSCs decreased after siSteap4 treatment, and the osteogenic capacity of the BMSCs was restored. The amount of Fe^2+^ was decreased and ferroptosis‐related proteins were also reduced by siSteap4 treatment. We also detected elevated expression of DMT1, which can transport Fe^2+^ into the cytoplasm. The above results showed knockdown of Steap4 can inhibit ferroptosis, alleviate senescence and enhance the osteogenic capacity in BMSCs. Considering the role of Steap4 in promoting ferroptosis in BMSCs, a novel murine model was used to conditionally knock down Steap4 in BMSCs using Prrx1‐Cre mice as previously described. The data showed knockdown of Steap4 using Prrx1‐Cre mice effectively reduces bone loss and delays the process of senile osteoporosis. In general, steap4 promotes the reduction of Fe^3+^ to Fe^2+^ in senescent BMSCs and that DMT1 transports Fe^2+^ into the cytoplasm, thereby inducing ROS damage and lipid peroxidation. Targeting Steap4 for the treatment of SOP has shown broad application prospects.

Interestingly, when the senescent BMSCs were treated with DFO, the expression of both Steap4 and DMT1 decreased. Therefore, we speculated that steap4 promotes Fe^2+^ accumulation in senescent BMSCs and that Fe^2+^ and ROS stimulate transcription factors to increase Steap4 expression, forming a positive feedback loop. Through transcription factor prediction methods and multiple experimental validations, we found that Fe^2+^ and ROS stimulate C/EBPβ to increase Steap4 expression, forming a positive feedback loop to promote cellular senescence. In glioblastoma, C/EBPβ responds to ROS and acts as a transcription factor, regulating the transcription of antioxidative reductases.^[^ ^28^ ^]^ Additionally, inhibition of C/EBPβ by activation of tropomyosin‐related kinase B receptor (TrkB) promotes bone formation, and C/EBPβ regulates BMSCs differentiation to adipocytes.^[^ ^29^, ^30^ ^]^ Thus, these results also indirectly support our conclusions that the Steap4/Fe^2+^‐ROS/C/EBPβ positive feedback loop plays an important role in osteoporosis.

However, Steap4 also has the ability to regulate copper balance. This part of this study was not complete and needs to be further refined by subsequent studies. In addition, senescent BMSCs can be transformed into a SASP and secrete a variety of cytokines and substances that affect other cellular activities in the bone marrow microenvironment.^[^ ^24^, ^31^ ^]^ Zhou et al. demonstrated that Steap4 promotes osteoclastogenesis by regulating cellular iron/ROS levels.^[^ ^14^ ^]^ In this study, we found that knockdown of Steap4 dramatically decreased the number of mature osteoclasts, which is not contradictory to its effects on osteoblasts. Interestingly, recent studies have shown that Steap4 contained in exosomes translocates into the bacterial cytoplasm and induces Fe^2+^ elevation, ultimately causing ferroptosis.^[^ ^15^ ^]^ Therefore, increased Steap4 in senescent BMSCs may be secreted into the bone marrow microenvironment to induce osteoclastogenesis, which requires further in‐depth research.

Finally, we further revealed that the Steap4/C/EBPβ axis is involved in adipogenesis and that DFO treatment effectively inhibited the Steap4/C/EBPβ axis‐induced adipogenesis in SOP mice. Steap4 is highly expressed in healthy adipose tissue and maintains healthy adipose tissue function.^[^ ^22^, ^23^ ^]^ Furthermore, C/EBPβ has the ability to activate the transcription of PPARg and C/EBPα, which in turn mediates the activation of adipocyte phenotype genes and promotes differentiation toward adipocytes.^[^ ^21^, ^32^ ^]^ Long‐term use of DFO may lead to eye diseases, systemic allergic reactions, and teratogenic effects.^[^ ^33^, ^34^, ^35^ ^]^ However, no obvious systemic side effects were observed in this study. Therefore, these results also indirectly support the finding that the Steap4/C/EBPβ axis is involved in adipogenesis and facilitates osteoporosis.

The study also has certain limitations. We have not verified these results using conditional gene knockout mice (Steap4 and Cebpb genes). It is hoped that subsequent studies will construct conditional gene knockout mice of the Steap4 and Cebpb genes for further research. Meanwhile, there was a lack of clinical data support. In the future, we will further conduct in‐depth research by integrating clinical data.

## 4 Conclusion

Overall, our study demonstrated, for the first time, that the Steap4/C/EBPβ axis promoted bone loss in SOP mice. Steap4 promotes Fe^2+^ accumulation in senescent BMSCs, and then, Fe^2+^ and ROS stimulate the transcription factor C/EBPβ to increase Steap4 expression, forming a positive feedback loop that ultimately accelerates ferroptosis and adipocyte differentiation in senescent BMSCs. Fortunately, DFO treatment reduced Fe^2+^ accumulation, attenuated ferroptosis and inhibited adipogenesis in senescent BMSCs, thereby mitigating bone loss in SOP mice. Therefore, Steap4 disrupts redox homeostasis in BMSCs and promotes BMSCs senescence in SOP, which provides evidence for the development of therapeutic strategies targeting SOP.

## 5 Experimental Section

### Animal Experiments

The C57BL/6 mice used in this study were all bred in a specific pathogen free (SPF)‐grade animal room. The living environment of the mice was set at a constant temperature of 22 ± 2 °C with a 12/12 h day/night cycle, and sufficient space was provided to allow free access to standard laboratory water and food. The animal experiments in this study were approved by the Animal Ethics Committee of Soochow University (SUDA20230221A01).

16‐week‐old C57BL/6 male mice were purchased from GemPharmatech (Nanjing, China), and the mice were kept for an additional 48 weeks. When the mice were aged, they were randomly assigned and used in subsequent trials. As previously described, all mice were housed in a SPF‐grade animal room under a 12‐h light/dark cycle at a temperature of 22 ± 2 °C and humidity of 50 ± 10%, with ad libitum access to standard laboratory diet and autoclaved water. The control group (or young group, 16‐week‐old C57BL/6 male mice) and aged group (64‐week‐old C57BL/6 male mice) received subcutaneous injections of sterile PBS twice a week for 8 weeks; the DFO group (DFO treatment of 64‐week‐old C57BL/6 male mice) received subcutaneous injections of 30 mg kg^−1^ DFO twice a week for 8 weeks.^[^ ^36^ ^]^

For knockdown of Steap4 in BMSCs, recombinant AAV‐U6‐MCS‐CMV‐Dio (Hanbio, Shanghai, China) vectors expressing a short hairpin RNA (shRNA) were used. Specifically, Prrx1‐Cre mice received intravenous administration of AAV vectors 1.71 × 10^12^ vg mL^−1^ carrying either AAV‐U6‐Dio‐shSteap4 or AAV‐U6‐Dio‐shNC (negative control). Prrx1‐Cre male mice were received tail intravenous injections of AAV vectors for the first time at the age of fourteen months. After two months, tail intravenous injections of AAV vectors were repeated. The mice were sacrificed at 18 months and specimens were harvested for further evaluation. The following sense strands were used: shSteap4 5′‐GAGCCTCCCTTCGGCTTATAT‐3′; shNC 5′‐TTCTCCGAACGTGTCACGTAA‐3′.

### Histological Examination

After bilateral femoral bone tissue was collected, the bone tissue was fixed, decalcified, paraffin‐embedded and sectioned. Then, the paraffin sections were dewaxed, rehydrated and stained with H&E and Masson to analyze the morphology of the bone tissue. Tissue staining was performed according to the instructions of an H&E staining kit (Beyotime Biotechnology, Shanghai, #C0105S) and a Masson staining kit (Yeasen Biotechnology, Shanghai, #60532ES74). Prussian blue staining was carried out to detect iron ions in the bone marrow of mice by a Prussian blue staining kit (Servicebio, Wuhan, #G1029). Additionally, ALP activity was assessed to observe the state of osteogenesis using BCIP/NBT ALP substrate (Yeasen Biotechnology, Shanghai, #36301ES01), and TRAP activity was assessed to observe the state of osteoclastogenesis using a TRAP assay kit (Beyotime Biotechnology, Shanghai, #P0332). All the stains were performed according to the instructions.

Also the specific markers in bone tissue were examined using IHC staining. Tissue cyclin‐dependent kinase inhibitor 2A (p16) (Proteintech, WuHan, #10883‐1‐AP), PPARG (Proteintech, WuHan, #16643‐1‐AP), OCN (ABclonal, WuHan, #A6205), Steap4 (Proteintech, WuHan, #11944‐1‐AP), solute carrier family 11 (proton divalent metal ion transporters) member 2 (Slc11a2 or DMT1) (Proteintech, WuHan, #20507‐1‐AP), and C/EBPβ (ABclonal, WuHan, #A0711) were detected. Briefly, the sections were rehydrated first, followed by heat‐induced antigen retrieval. Then, 3% H_2_O_2_ was added to the sections for 10 min to eliminate the inner peroxidase, and goat serum was used to block the sections for 1 h. Primary antibodies, including anti‐p16 (1:1000), anti‐PPARG (1:200), anti‐OCN (1:100), anti‐Steap4 (1:100), anti‐DMT1 (1:100) and anti‐C/EBPβ (1:100), were added to the sections for incubation overnight at 4 °C. Finally, an ABC kit (Vector) and diaminobenzidine (Sigma‒Aldrich) were used for staining.

### Micro‐CT Analysis

To evaluate the bone structure and bone mineral density, micro‐CT (Bruker SkyScan 1174, Belgium) was used in this study. The parameters were set as follows: filter Al 0.5 mm, source voltage (kV) 50, source current 500 µA, and scanning layer thickness 9 µm. Other parameters were measured as previously described.^[^ ^37^ ^]^

### Cell Culture

After C57BL/6 mice were sacrificed via cervical dislocation, they were quickly immersed in 75% ethanol for 5 min. Then, the bilateral femurs and tibiae were removed into an ultraclean bench that had been sterilized in advance, and the excess muscles and ligaments were removed as much as possible. Next, the tissues were sequentially soaked and sterilized with 75% ethanol, sterile PBS, 75% ethanol and sterile PBS for 1 min each time to achieve adequate disinfection. The serum iron levels and the bone marrow cavity iron levels in mice were detected by a iron determination kit (Jiancheng Bioengineering Institute, Nanjing, #A039‐1‐1). The bone marrow cavity was exposed and placed in prewarmed α‐minimum essential medium (MEM) medium containing 20% FBS at 37 °C. The bone marrow cavity was repeatedly rinsed with a syringe until the bone turned white, and the supernatant was discarded after centrifugation at 1200 rpm for 3 min. The erythrocytes were lysed by adding erythrocyte lysis solution, left at room temperature for 3 min, and centrifuged at 1200 rpm for 3 min, after which the supernatant was discarded. Finally, the harvested cells were resuspended in α‐MEM containing 20% fetal bovine serum (FBS) and seeded in 10‐cm cell culture dishes. After 48 h of culture in a cell incubator with 5% CO_2_ at 37 °C, the medium was completely changed, and the adherent cells were considered BMSCs. The medium was changed every three days, and the BMSCs were passaged to the second generation and used for subsequent experiments. For in vitro experiments, BMSCs extracted from 64‐week‐old C57BL/6 male mice were used as Senescent group, and BMSCs extracted from 16‐week‐old C57BL/6 male mice were used as Control group.

C57/BL6 mouse femurs and tibiae were used to extract primary bone marrow macrophages (BMMs). The cells were cultured in dulbecco's modified eagle medium (DMEM) containing 100 U mL^−1^ penicillin, 10% FBS and macrophage colony‐stimulating factor (M‐CSF) (30 ng mL^−1^). After 24 h, unattached cells were removed and adherent cells were cultured for another 72 h. In addition, For osteoclast differentiation, BMMs were cultured in induction medium containing 100 ng mL^−1^ receptor activator of nuclear factor‐rB ligand (RANKL). After 5 d osteoclast induction, TRAP staining was performed following standard procedures. The multinucleated cells were observed under an inverted optical microscope.

### Osteogenic Induction Culture

BMSCs osteogenic differentiation was induced using osteogenic induction medium. The formulation of osteogenic induction medium used in this experiment was as follows: 10% FBS, 1% P/S, 50 µg mL^−1^ vitamin C, 10 mm β‐glycerophosphate and 10 nm dexamethasone were added to the α‐MEM. Dexamethasone and vitamin C were stored in the dark. BMSCs were seeded in well plates, and osteogenic induction medium was added when the cell density reached 80%. The medium was changed every 3 days, and the culture was terminated at different time points depending on the experiment. Then, the cells were harvested for different experiments.

### ARS Staining and ALP Staining

In this study, 4–5 × 10^4^ BMSCs were seeded in 24‐well plates in each well for ALP and ARS assays. Osteogenic induction was started when the cell density reached 80%, and the osteogenic medium was changed every 3 days. ALP staining was performed after 14 days of osteogenic induction. ARS staining was subsequently performed on day 21. After induction, the BMSCs were washed three times with precooled 4 °C PBS for 5 min each time and fixed in 4% paraformaldehyde for 15 min. Then, the cells were stained according to the instructions of the ALP and ARS staining kits (Yeasen Biotechnology, Shanghai, #36301ES01 and #60504ES25). In addition, the enzyme activity of ALP was quantified using an ALP activity detection kit (Elabscience, Wuhan, #E‐BC‐K009‐M). Cetylpyridinium chloride (10%) was used to dissolve the ARS‐mineralized nodules, and the optical density (OD) of the solution was measured at 570 nm for quantitative analysis of the resulting ARS staining.

### Cellular Senescence Detection by β‐Gal Staining

BMSCs were cultured in a 12‐well plate with 7 × 10^5^ cells per well. After 24 h of culture, the BMSCs in the different groups were subjected to different treatments. Finally, the medium was removed, and the BMSCs were rinsed with prechilled PBS and then fixed with 4% paraformaldehyde. A working solution from a senescence β‐Gal staining kit (Beyotime Biotechnology, Shanghai, #C0602) was added to BMSCs for 10 h at 37 °C.

### RNA Sequencing

In this study, BMSCs were divided into three groups for RNA sequencing: 1) BMSCs from 4‐month‐old mice were treated with osteogenic medium for 3 days, 2) BMSCs from 16‐month‐old mice were treated with osteogenic medium for 3 days, and 3) BMSCs from 16‐month‐old mice were treated with osteogenic medium and DFO for 3 days. After intervention, the cells were quickly washed with precooled PBS at 4 °C and lysed with TRIzol; then, the cells were harvested and placed in liquid nitrogen for quick freezing to prevent RNA degradation. Subsequent RNA extraction, RNA sample quality control, library construction, library purification, library detection, library quantification, generation of sequencing clusters, and computer sequencing were all performed at AZENTA Biotechnology, Inc. (Suzhou). The transcriptome sequencing data used in this study were generated based on the Illumina sequencing platform.

### Detection of Ferroptosis

Ferroptosis is an iron‐dependent form of cell death characterized by the accumulation of iron‐dependent lipid peroxidation products that eventually reach deadly levels. Therefore, the following factors were selected for ferroptosis assessment: total cellular ROS, lipid ROS, lipid peroxidation, iron ions, ferrous ions in mitochondria and changes in the mitochondrial membrane potential. A ROS assay kit (Beyotime Biotechnology, Shanghai, #S0033M) was used to detect cellular ROS; lipid ROS levels were measured using the Bodipy 581/591 C11 fluorescent probe (DOJINDO, Japan, #L267) following the manufacturer's protocol; a Bodipy 581/591 C11 fluorescent probe (ABclonal Technology, WuHan, #RM02821) was used to detect lipid peroxidation; a FerroOrange fluorescent probe (DOJINDO, Japan, #F374) was used to detect intracellular iron ions; a Mito‐FerroGreen fluorescent probe (DOJINDO, Japan, #M489) was used to detect ferrous ions and a JC‐1 mitochondrial membrane potential (MitoMP) detection kit (DOJINDO, Japan, #MT09) was used to assess mitochondrial function. When using ROS and JC‐1 reagents to measure the levels of ROS and MitoMP, not only fluorescence microscopy was used for photographic evaluation but also flow cytometry to quantify the average fluorescence intensity.

### RT‒qPCR

Total cellular RNA was extracted with RNA extraction reagent (Yeasen Biotechnology, Shanghai, 19202ES60) according to the manufacturer's instructions. An appropriate amount of RNase‐free ddH2O was added to the extracted total RNA. The RNA concentration and the OD260/OD280 ratio were detected using a Nanodrop2000 (Thermo Fisher, USA). RNA with OD ratios between 1.8 and 2.2 were used for subsequent RT‒qPCR assays. An RNA reverse transcription kit (Yeasen Biotechnology, Shanghai, #11151ES60) was used for reverse transcription, and SYBR Green (Yeasen Biotechnology, Shanghai, #11204ES50) was used for RT‒qPCR. The sequences of primers used were as follows:

***Steap4***

Forward primer GGGAAGTCACTGGGATTGAAAA;

Reverse primer CCGAATAGCTCAGGACCTCTG.

***Dmt1***

Forward primer CAATGTCTTTGTCGTGTCCGT;

Reverse primer GCGACCATTTTAGGTTCAGGAAT.

***Cebpb***

Forward primer CGCCGCCTTATAAACCTCCC;

Reverse primer AGTCGGGCTCGTAGTAGAAGT.

***Pparg***

Forward primer TCGCTGATGCACTGCCTATG;

Reverse primer GAGAGGTCCACAGAGCTGATT.

***Fasn***

Forward primer GGAGGTGGTGATAGCCGGTAT;

Reverse primer TGGGTAATCCATAGAGCCCAG.

***Fabp4***

Forward primer AAGGTGAAGAGCATCATAACCCT;

Reverse primer TCACGCCTTTCATAACACATTCC.

***Runx2***

Forward primer TTGACCTTTGTCCCAATGC;

Reverse primer AGGTTGGAGGCACACATAGG.

***Osterix***

Forward primer TGAGCTGGAACGTCACGTGC;

Reverse primer AAGAGGAGGCCAGCCAGACA.

***Ocn***

Forward primer TCCCACACAGCAGCTTGGCCC;

Reverse primer TGAGGCTCCAAGGTAGCGCCG.

### Western Blot Analysis

Radio immunoprecipitation assay (RIPA) lysis buffer (Yeasen Biotechnology, Shanghai, #20115ES60) was used to lyse cells and tissue samples, and protease inhibitors were also purchased from Yeasen Biotechnology (#20123ES10). The protein concentrations were detected with a BCA Protein Quantification Kit (Yeasen Biotechnology, #20201ES86). The samples were subjected to 8%, 10%, or 12% SDS‐PAGE gel to separate proteins with different molecular weights. The separated proteins were transferred to polyvinylidene fluoride (PVDF) 0.45 µm membranes (Millipore). 5% nonfat milk was used to block the PVDF membrane for 1 h, and the membranes were incubated with primary antibodies, including anti‐Steap4 (Proteintech, WuHan, #11944‐1‐AP), anti‐C/EBPβ (Proteintech, WuHan, #23431‐1‐AP), anti‐FTL (Proteintech, WuHan, #10727‐1‐AP), anti‐GPX4 (ABclonal, WuHan, #A1933), anti‐FTH1 (ABclonal, WuHan, #A19544), anti‐NCOA4 (ABclonal, WuHan, #A5695), anti‐TFRC (ABclonal, WuHan, #A22161), anti‐DMT1 (ABclonal, WuHan, #A10231), anti‐Runx2 (ABclonal, WuHan, #A2851), anti‐Osterix (ABclonal, WuHan, #A18699), anti‐OCN (ABclonal, WuHan, #A6205), and anti‐β‐actin (Proteintech, WuHan, #81115‐1‐RR), at 4 °C with gentle shaking overnight. The dilution ratio of anti‐C/EBPβ to anti‐β‐actin was 1:5000, and the dilution ratio of anti‐steap4 was 1:1000. Horseradish peroxidase‐conjugated anti‐rabbit IgG secondary antibody (YEASEN) was used in this study, and after incubation with the secondary antibody, the PVDF membranes were subjected to electrochemiluminescence (YEASEN) and detected using a ChemiDoc XRS+ system (Bio‐Rad).

### RNA Interference and Overexpression

To inhibit Steap4 and C/EBPβ expression, BMSCs were transfected with small interfering RNA (siRNA). The siRNA target sequence was designed in Invitrogen Block‐iT RNAi designer (Thermo Fisher). Two siRNAs were designed for Steap4 and C/EBPβ:

 siSteap4 (1) target DNA sequence: CCTCTGACTACTGATTCCTCAGAAA

 sense sequence: CCUCUGACUACUGAUUCCUCAGAAA

 antisense sequence: UUUCUGAGGAAUCAGUAGUCAGAGG

 siSteap4 (2) target DNA sequence: GAGCTATTCGGAAGCAGCATCCAAG

 sense sequence GAGCUAUUCGGAAGCAGCAUCCAAG

 antisense sequence CUUGGAUGCUGCUUCCGAAUAGCUC

The siSteap4 (negative control) target DNA sequence was CCTAGTCCAGTTACTTCACGTCAAA

 sense sequence CCUAGUCCAGUUACUUCACGUCAAA

The antisense sequence UUUGACGUGAAGUAACUGGACUAGG

 siCebpb (1) target DNA sequence: AGACCCATGGAAGTGGCCAACTTCT

 sense sequence: AGACCCAUGGAAGUGGCCAACUUCU

 antisense sequence: AGAAGUUGGCCACUUCCAUGGGUCU

 siCebpb (2) target DNA sequence: CCCATGGAAGTGGCCAACTTCTACT

 sense sequence: CCCAUGGAAGUGGCCAACUUCUACU

 antisense sequence: AGUAGAAGUUGGCCACUUCCAUGGG

 siCebpb (negative control) target DNA sequence: CCCGGAATGGGCCAATCCTTTAACT

 sense sequence: CCCGGAAUGGGCCAAUCCUUUAACU

 antisense sequence: AGUUAAAGGAUUGGCCCAUUCCGGG

After incubation with siRNA for 36 h, the cells were incubated with normal medium for 24 h and collected for western blot analysis to detect knockdown inefficiencies.

### Adipogenic Induction

BMSC adipose differentiation was induced using adipogenic induction medium. The formulation of adipogenic induction medium used in this experiment was as follows: adipogenic medium (A) was composed of 10% FBS, 1% P/S, 500 µm isobutylmethylxanthine (IBMX), 5 µg ml^−1^ insulin, 200 µm indomecin, and 150 nm dexamethasone phosphate disodium in α‐MEM, and adipogenic medium (B) was composed of 10% FBS, 1% P/S, and 5 µg ml^−1^ insulin in α‐MEM. BMSCs were seeded in well plates, and adipogenic medium (A) was added when the cell density reached 80%. After 72 h of induction with adipogenic medium (A), the culture medium was replaced with adipogenic medium (B) for 72 h. Then, the medium was changed every 3 days, and the culture continued until lipid droplets appeared. Then, the cells were harvested for different experiments.

### Oil Red‐O Staining

Lipid droplets were analyzed by oil red‐O staining. BMSCs were fixed with 10% paraformaldehyde for 15 min, washed with precooled PBS and stained with oil red‐O (Beyotime Biotechnology, Shanghai, #C0157S) solution for 20 min.

### Immunofluorescence Assay

Immunofluorescence assay was conducted to study the expression of aging‐related marker proteins in BMSCs. Cells were added to a 24‐well plate (5 × 10^4^ cells per well). After different treatment, cells were fixed and washed with sterile PBS. The nonspecific binding sites of the cells were blocked with 2% BSA‐PBS. The fixed cells were incubated at 4 °C with antibodies of p21 (ABclonal, WuHan, #A22460PM), p53 (ABclonal, WuHan, #A25915) and p16 (Thermo Fisher, Shanghai, #PA5‐20379) for 12 h. After that, cells were incubated with fluorescent‐labeled secondary antibodies for 15 min at 37 °C. Images were captured with an immunofluorescence microscope.

### ChIP

ChIP kit (Thermo Fisher, Shanghai, #26 156) was used for ChIP assay following manufacturer's protocol. In brief, cells were fixed with formaldehyde to promote cross‐linking of proteins to chromatins. The fragmented chromatins (200–1000 bp) were pre‐cleared to remove non‐specific binding, followed by overnight incubation with a Cebpb‐specific antibody (Proteintech, WuHan, # 23431‐1‐AP) to induce the formation of immune complexes. IgG was used for negative control. These complexes were immunoprecipitated by Protein A agarose beads. Then the precipitated complex was cleaned and some non‐specific binding was removed by elution. After that, the cross‐links were reversed, proteins were digested, and the purified DNA was obtained for qPCR analysis. The three qPCR primers were as follows:

Site1 Forward primer: GTCAGAATGTCTGCTGCCCT Reverse primer: GATCGGGACTTTAGAGCGCA, product size 207 bp;

Site2 Forward primer: TGAAGACCTGAAAACTCTCCATCT Reverse primer: ACATGGGCAAGGAGCTTAGG, product size 132 bp;

Site3 Forward primer: CCAGGTTGAACTCTTCCCGG Reverse primer: TCCAGGCTCCCGCTTTTAAG, product size 101 bp.

### Statistical Analyses

All the statistical analyses were performed using GraphPad Prism version 9.0. The mean ± standard deviation was used to describe continuous variable data. Categorical variable data were expressed as frequencies and proportions. The homogeneity of variance test and one‐way analysis of variance (ANOVA) were performed to determine whether the difference between groups was statistically significant. According to the suggestion of GraphPad Prism 9.0, after one‐way ANOVA, Dunnett's multiple comparison test was chosen as the post hoc test. For the data that were not suitable for the parametric test, the nonparametric Kruskal‒Wallis test was used. *P* < 0.05 was considered to indicate statistical significance.

## Conflict of Interest

The authors declare no conflicts of interest.

## Supporting information



Supporting Information

## Data Availability

The data that support the findings of this study are available from the corresponding author upon reasonable request.
