# Supplementary material for: Steap4 Promotes Senile Osteoporosis via Fe2+‐ROS/C/EBPβ Feedback‐Driven Ferroptosis and Adipogenesis in Senescent BMSCs
Source: Adv Sci (Weinh). 2025 Nov 7;13(5):e09926. doi: 10.1002/advs.202509926 (PMC12850026; doi:10.1002/advs.202509926)
Supplement: Supplementary file 1 — Supporting Information [file ADVS-13-e09926-s001.docx]

**Supporting Information**

**Steap4 promotes senile osteoporosis via Fe²⁺-ROS/C/EBPβ feedback-driven ferroptosis and adipogenesis in senescent BMSCs**

**The name(s) of the author(s):**

Liangliang Wang^1,†^; Guangrong Yin^1,†^; Wenming Li^2,†^; Maoyuan Li^1^; Feng Lu^1^; Chao Xu^1^; Gongyin Zhao^1^; Dechun Geng^2*^; Jiaxiang Bai^3*^; Yuji Wang^1,4*^

**Corresponding authors:**

**Dechun Geng**

Department of Orthopaedics, The First Affiliated Hospital of Soochow University, Suzhou, 215006, China.

E-mail: **szgengdc@suda.edu.cn**;

**Jiaxiang Bai**

Department of Orthopedics, Centre for Leading Medicine and Advanced Technologies of IHM, The First Affiliated Hospital of USTC, Division of Life Sciences and Medicine, University of Science and Technology of China, Hefei, 230022, China.

E-mail: **[jxbai1995@ustc.edu.cn](mailto:jxbai1995@ustc.edu.cn)**;

**Yuji Wang**

Department of Orthopaedics, the Affiliated Changzhou Second People’s Hospital of Nanjing Medical University, Changzhou Medical Center, Nanjing Medical University, Changzhou, 213003, China.

E-mail: **yujiwang1036@njmu.edu.cn**.

^†^ **Contribute equally to this work**


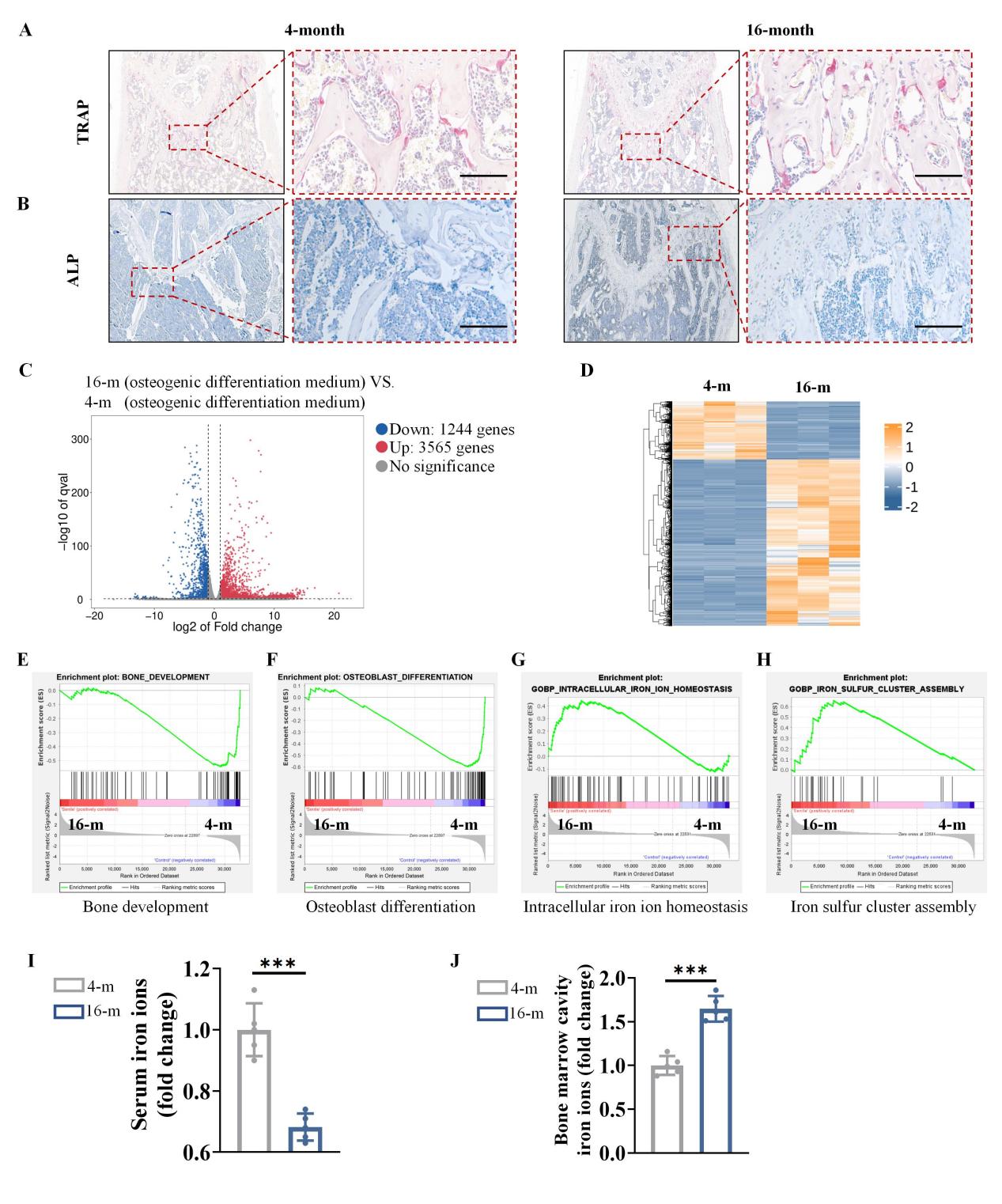


**Supplementary Figure S1** (A) Osteoclast TRAP staining (scale bar: 100 μm). (B) Osteoblast ALP staining (scale bar: 100 μm). RNA sequencing analysis with BMSCs from 4-month-old mice were treated with osteogenic medium for 3 days, and BMSCs from 16-month-old mice were treated with osteogenic medium for 3 days. (4-m: 4-month-old; 16-m: 16-month-old.) (C) Volcano plot. (D) Heat map. (E) (F) (G) (H) GSEA. (I) Serum iron levels (n=5). (J) bone marrow cavity iron levels (n=5). (The data are presented as the means ± SDs. Statistical analysis was performed using one-way ANOVA. ***p < 0.001, **p < 0.01, *p < 0.05.)


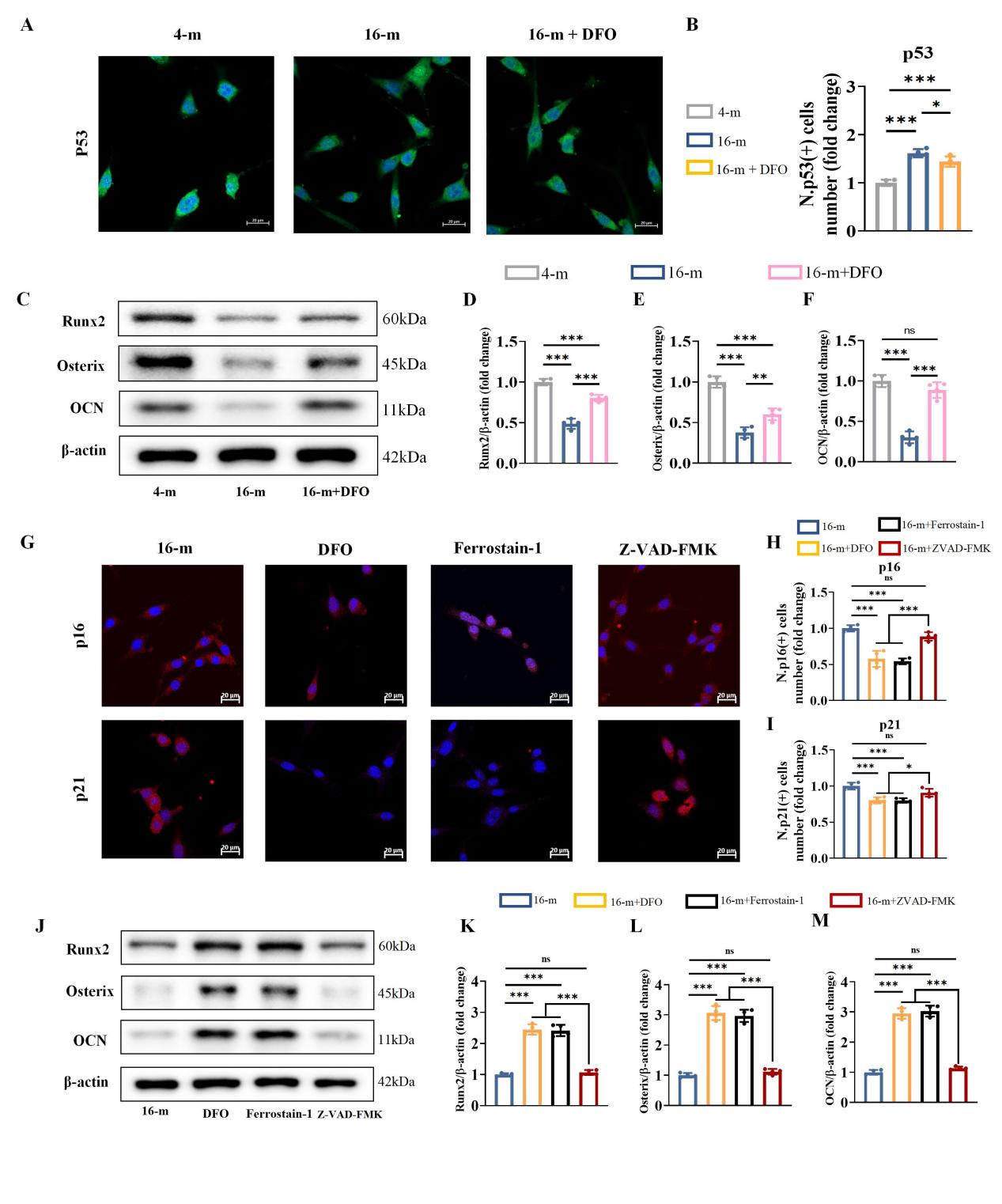


**Supplementary Figure S2** (A) Immunofluorescence staining of p53 (green for p53 and blue for nuclei, scale bar: 20 μm). (B) Quantitative analysis of p53. (C) Runx2, Osterix and OCN protein levels, detected by western blot analysis. Quantitative analysis of (D) Runx2, (E) Osterix and (F) OCN. (G) Immunofluorescence staining of p16 (red for p16 and blue for nuclei, scale bar: 20 μm) and p21 (red for p21 and blue for nuclei, scale bar: 20 μm). Quantitative analysis of (H) p16 and (I) p21. (J) Runx2, Osterix and OCN protein levels, detected by western blot analysis. Quantitative analysis of (K) Runx2, (L) Osterix and (M) OCN. (The data are presented as the means ± SDs. Statistical analysis was performed using one-way ANOVA. ***p < 0.001, **p < 0.01, *p < 0.05, n=4.)


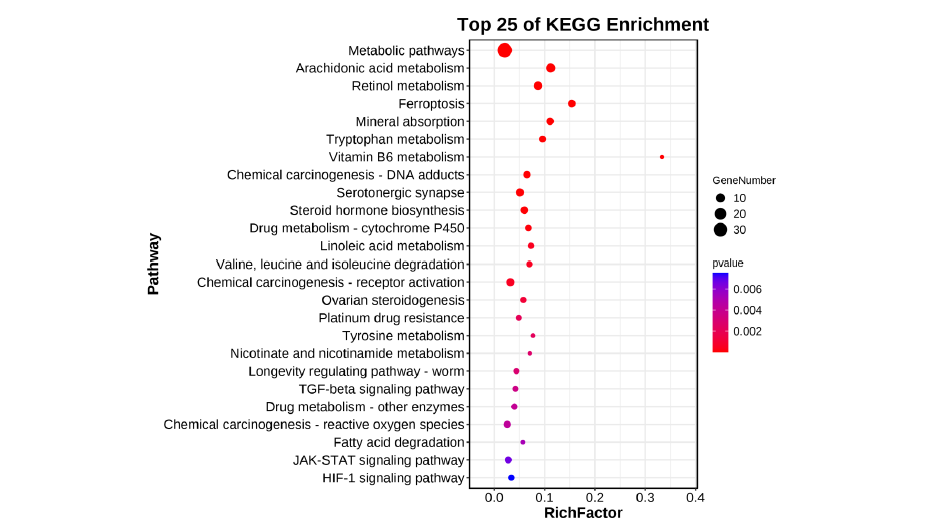


**Supplementary Figure S3** KEGG enrichment analysis on 78 DEGs from Figure 3A.


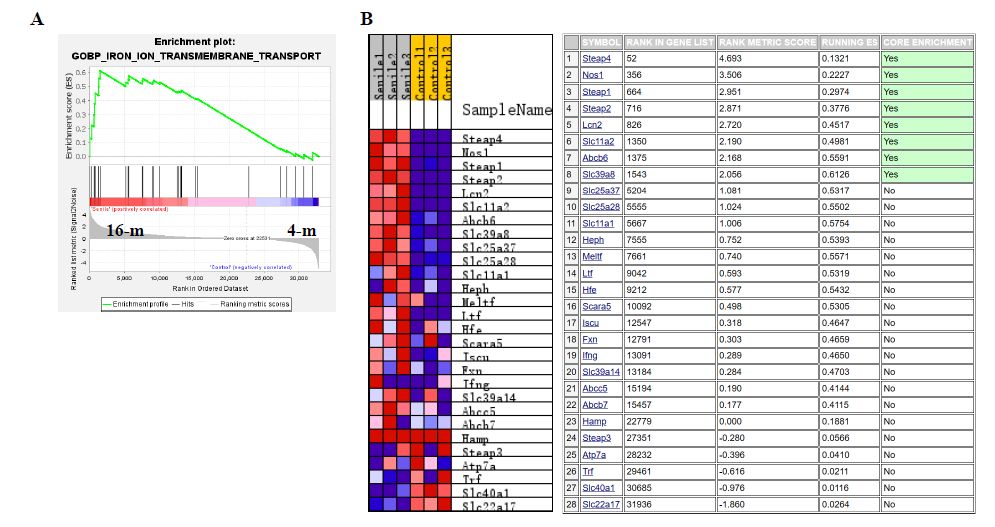


**Supplementary Figure S4** GSEA analysis on transmembrane transport process.


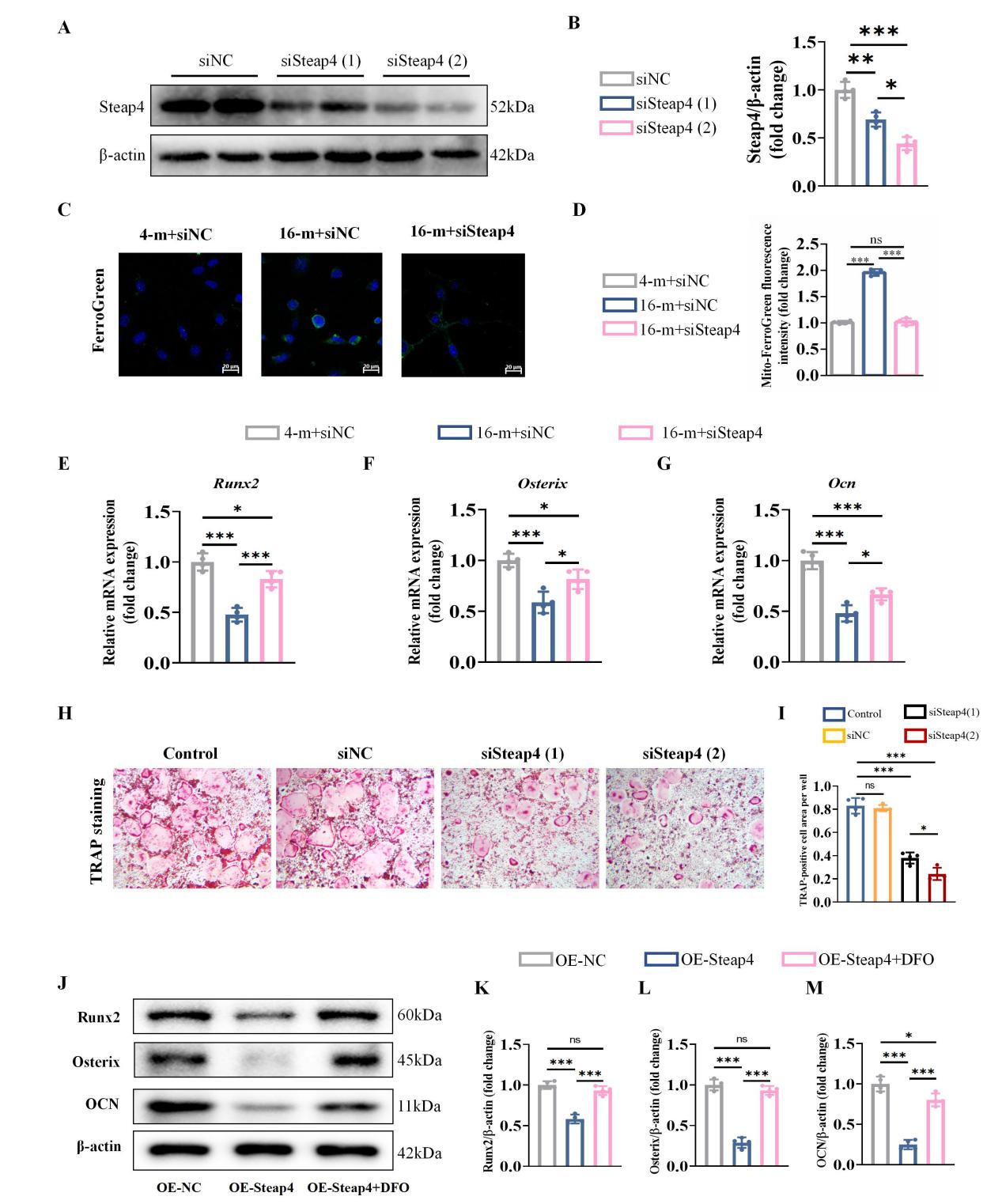


**Supplementary Figure S5** (A) Steap4 protein level, detected by western blot analysis. (B) Quantitative analysis of Steap4. (C) FerroGreen Assay (scale bar: 20 μm). (D) Quantitative analysis of FerroGreen. RT‒qPCR of (E) *Runx2*, (F) *Osterix* and (G) *Ocn*. (H) TRAP staing: knockdown of Steap4 dramatically decreased the number of mature osteoclasts. (I) Quantitative analysis of TRAP-positive cell area per well. (J) Runx2, Osterix and OCN protein levels, detected by western blot analysis. Quantitative analysis of (K) Runx2, (L) Osterix and (M) OCN. (The data are presented as the means ± SDs. Statistical analysis was performed using one-way ANOVA. ***p < 0.001, **p < 0.01, *p < 0.05, n=4.)

**
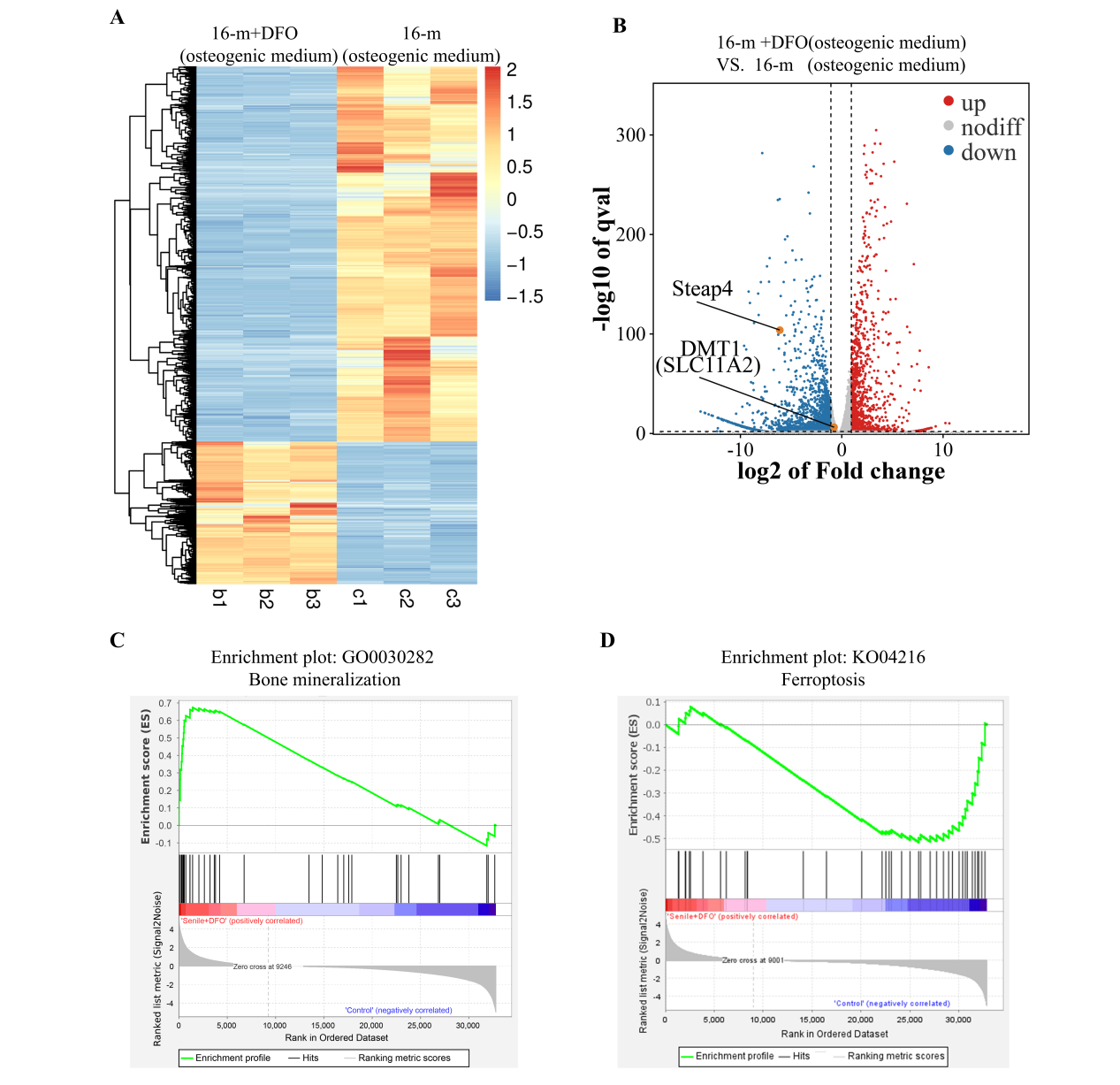
**

**Supplementary Figure S6** (A) Heatmap between senescent BMSCs and DFO-treated senescent BMSCs. (B) Volcano plot. (BMSCs from 16-month-old mice were treated with osteogenic medium for 3 days, and BMSCs from 16-month-old mice were treated with osteogenic medium and DFO for 3 days.) (C) (D) GSEA.


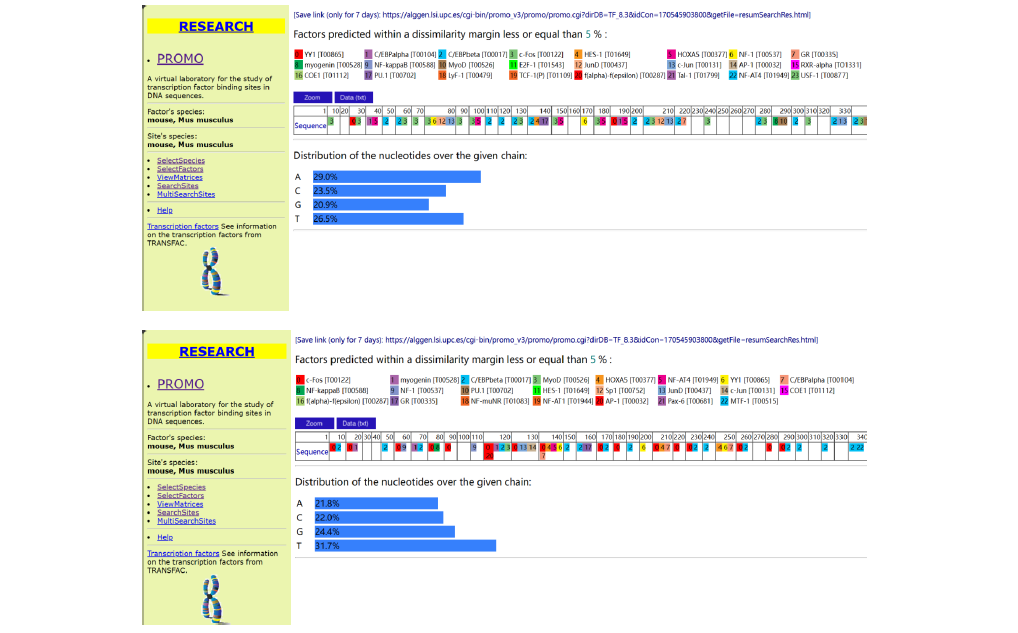


**Supplementary Figure S7** The transcription factor-binding site prediction website (PROMO) was used to detect the transcription factors of Steap4 and DMT1 in promoter regions.


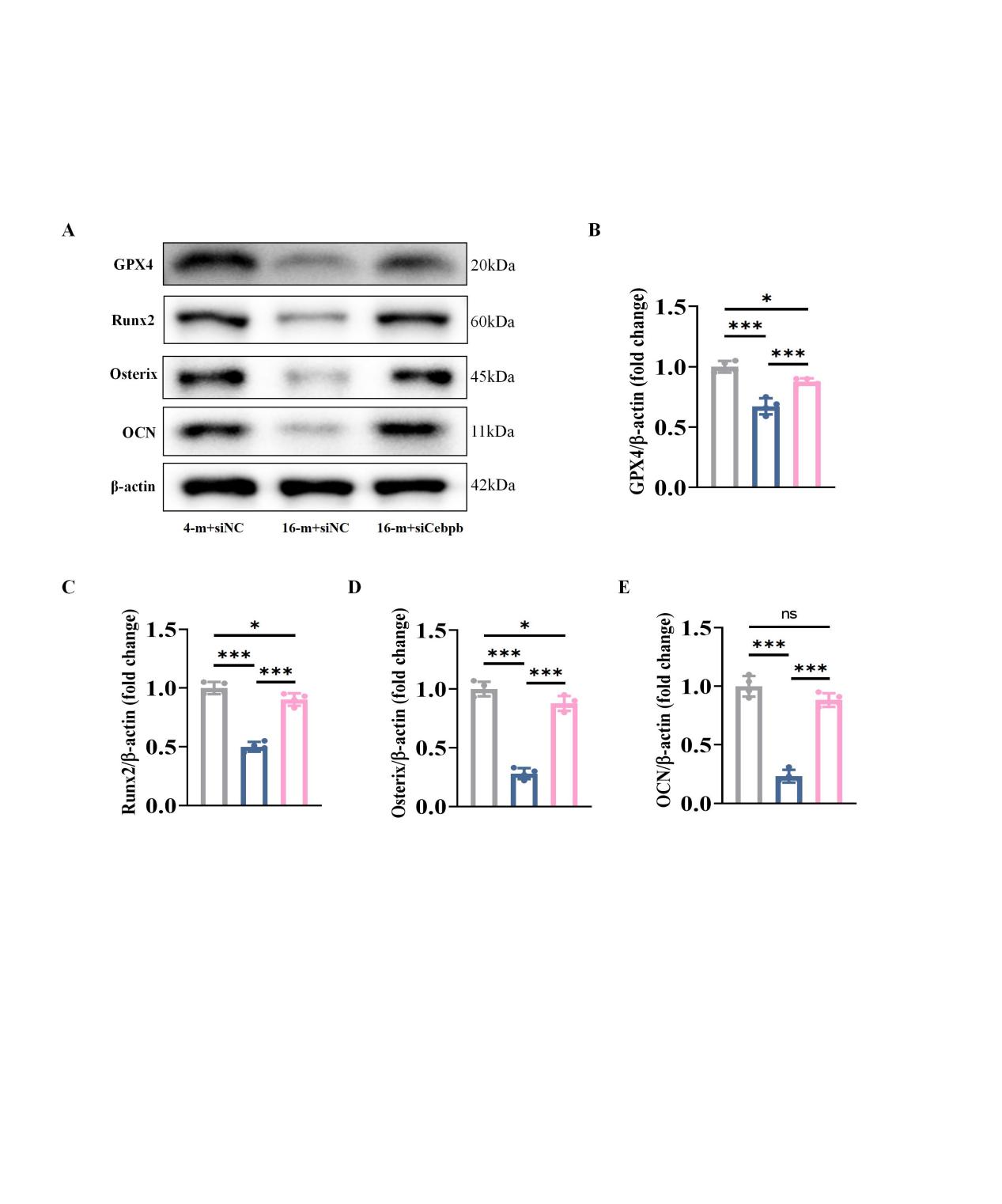


**Supplementary Figure S8** (A) GPX4, Runx2, Osterix and OCN protein levels, detected by western blot analysis. Quantitative analysis of (B) GPX4, (C) Runx2, (D) Osterix and (E) OCN. (The data are presented as the means ± SDs. Statistical analysis was performed using one-way ANOVA. ***p < 0.001, **p < 0.01, *p < 0.05, n=4.)


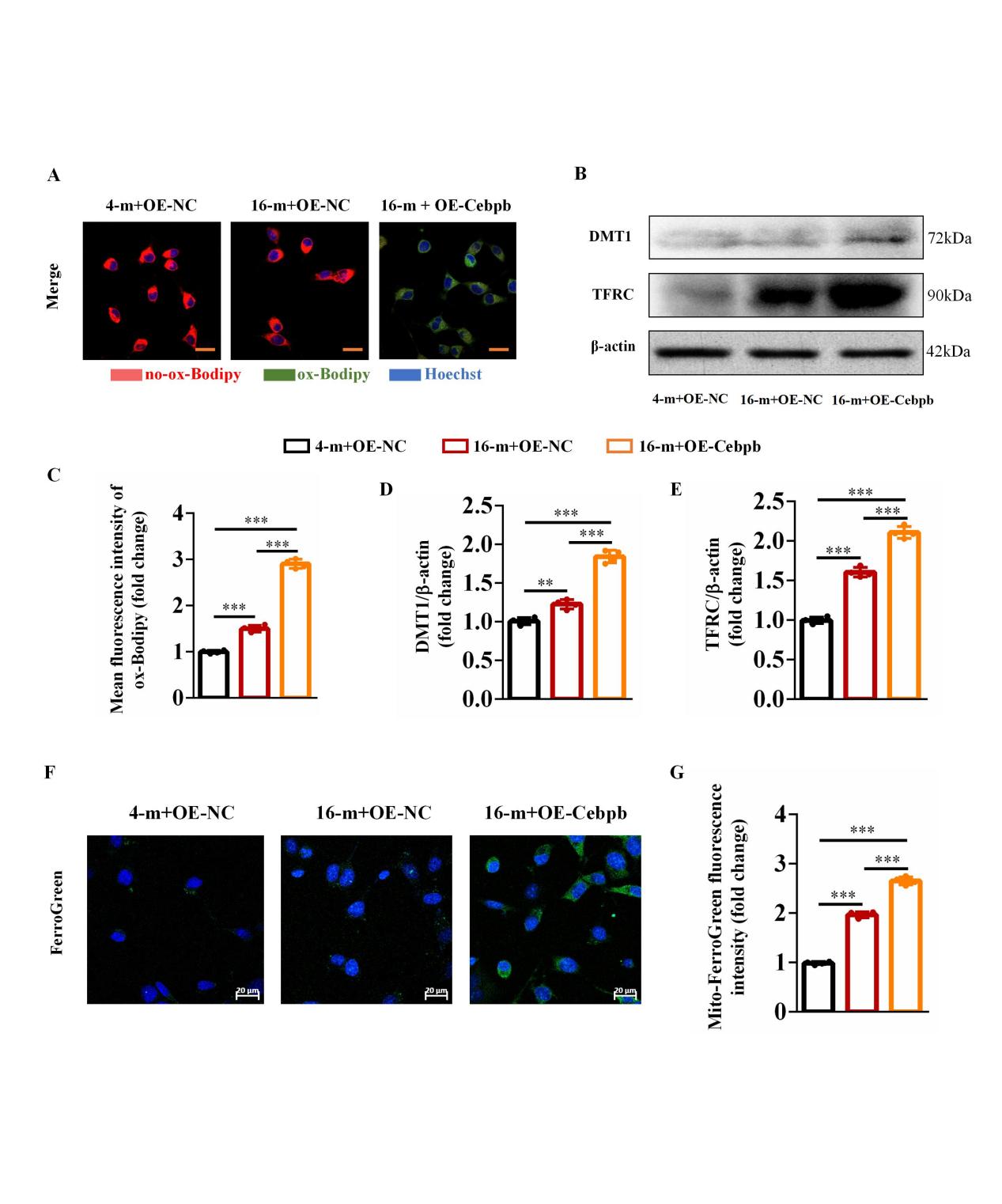


**Supplementary Figure S9** (A) Images of the BMSCs after coimmunostaining: red (no-ox-Bodipy for non-lipid peroxidation), green (ox-Bodipy for lipid peroxidation) and blue (hoechst, scale bar: 20 μm). (B) DMT1 and TFRC protein levels, detected by western blot analysis. (C) Quantitative analysis of ox-Bodipy. (D) Quantitative analysis of DMT1. (E) Quantitative analysis of TFRC. (F) FerroGreen Assay (scale bar: 20 μm). (G) Quantitative analysis of FerroGreen. (The data are presented as the means ± SDs. Statistical analysis was performed using one-way ANOVA. ***p < 0.001, **p < 0.01, *p < 0.05, n=4.)


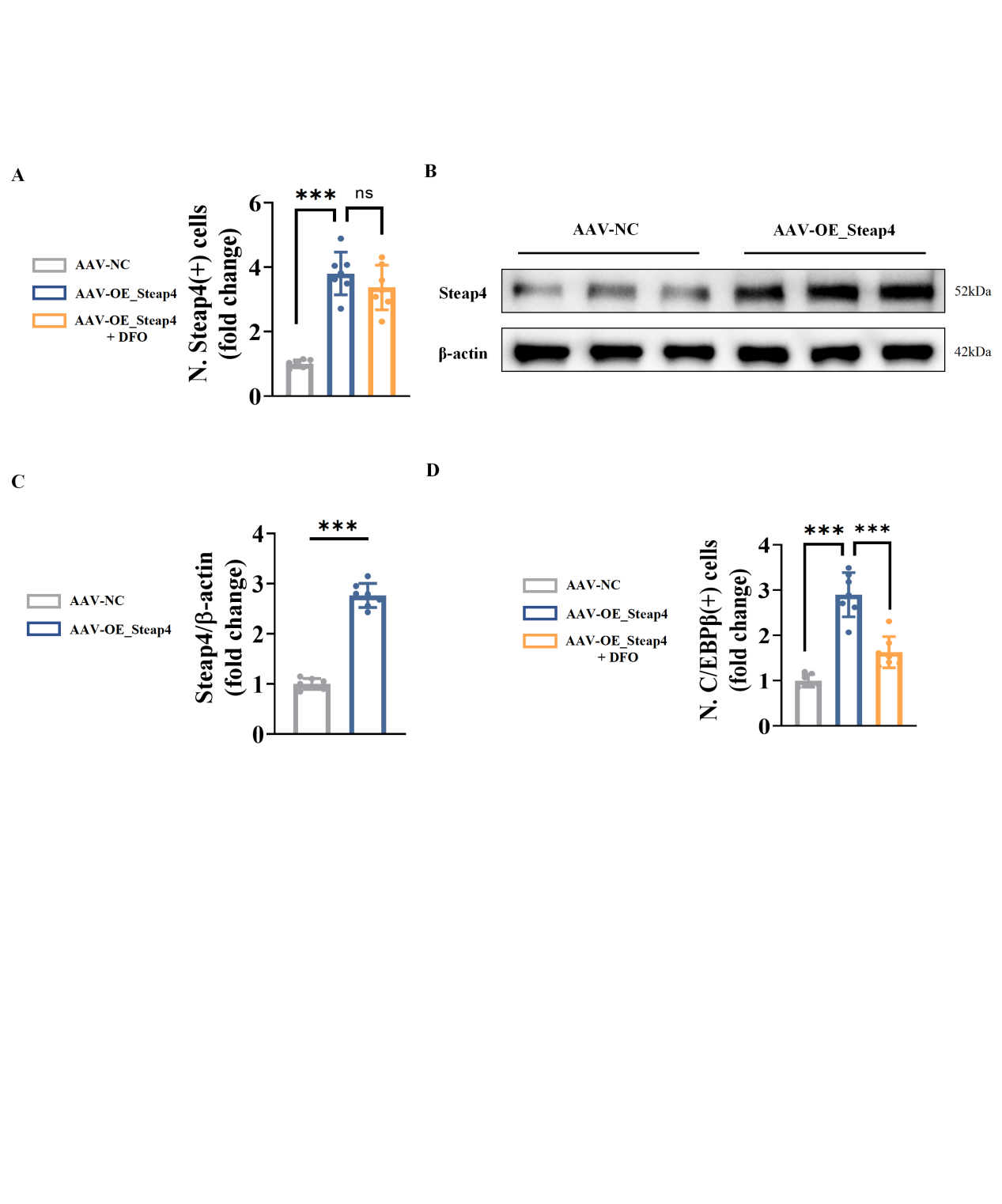


**Supplementary Figure S10** (A) Quantitative analysis of Steap4(+) cells. (B) Steap4 protein levels, detected by western blot analysis. (C) Quantitative analysis of Steap4. (D) Quantitative analysis of C/EBPβ(+) cells. (The data are presented as the means ± SDs. Statistical analysis was performed using one-way ANOVA. ***p < 0.001, **p < 0.01, *p < 0.05, n=7.)

| **Table S1. 78 DEGs in the Venn analysis in Figure 3A** | |
| --- | --- |
| gene_id | gene_name |
| ENSMUSG00000024140 | Epas1 |
| ENSMUSG00000026822 | Lcn2 |
| ENSMUSG00000017969 | Ptgis |
| ENSMUSG00000001751 | Naglu |
| ENSMUSG00000012428 | Steap4 |
| ENSMUSG00000024066 | Xdh |
| ENSMUSG00000015653 | Steap2 |
| ENSMUSG00000023191 | P3h3 |
| ENSMUSG00000028641 | P3h1 |
| ENSMUSG00000019055 | Plod1 |
| ENSMUSG00000021109 | Hif1a |
| ENSMUSG00000055932 | Fto |
| ENSMUSG00000051048 | P4ha3 |
| ENSMUSG00000032340 | Neo1 |
| ENSMUSG00000033022 | Cdo1 |
| ENSMUSG00000057329 | Bcl2 |
| ENSMUSG00000020641 | Rsad2 |
| ENSMUSG00000022797 | Tfrc |
| ENSMUSG00000023030 | Slc11a2 |
| ENSMUSG00000042599 | Kdm7a |
| ENSMUSG00000078597 | Cyp4a12b |
| ENSMUSG00000020649 | Rrm2 |
| ENSMUSG00000079057 | Cyp4v3 |
| ENSMUSG00000006818 | Sod2 |
| ENSMUSG00000029716 | Tfr2 |
| ENSMUSG00000021048 | Mthfd1 |
| ENSMUSG00000038644 | Pold1 |
| ENSMUSG00000019841 | Rev3l |
| ENSMUSG00000056962 | Jmjd6 |
| ENSMUSG00000025002 | Cyp2c55 |
| ENSMUSG00000074183 | Gsta1 |
| ENSMUSG00000006764 | Tph2 |
| ENSMUSG00000024661 | Fth1 |
| ENSMUSG00000062168 | Ppef1 |
| ENSMUSG00000022199 | Slc22a17 |
| ENSMUSG00000053897 | Slc39a8 |
| ENSMUSG00000029246 | Ppat |
| ENSMUSG00000025508 | Rplp2 |
| ENSMUSG00000023963 | Cyp39a1 |
| ENSMUSG00000029361 | Nos1 |
| ENSMUSG00000029727 | Cyp3a13 |
| ENSMUSG00000005413 | Hmox1 |
| ENSMUSG00000032012 | Nectin1 |
| ENSMUSG00000039183 | Nubp2 |
| ENSMUSG00000064254 | Ethe1 |
| ENSMUSG00000015652 | Steap1 |
| ENSMUSG00000026198 | Abcb6 |
| ENSMUSG00000020892 | Aloxe3 |
| ENSMUSG00000038656 | Cyp3a16 |
| ENSMUSG00000022346 | Myc |
| ENSMUSG00000051232 | Tmem199 |
| ENSMUSG00000021340 | Gpld1 |
| ENSMUSG00000038403 | Hjv |
| ENSMUSG00000028713 | Cyp4b1 |
| ENSMUSG00000032496 | Ltf |
| ENSMUSG00000031936 | Hephl1 |
| ENSMUSG00000040121 | Rep15 |
| ENSMUSG00000064294 | Aox3 |
| ENSMUSG00000038242 | Aox4 |
| ENSMUSG00000075604 | Cyp11b1 |
| ENSMUSG00000049287 | Iba57 |
| ENSMUSG00000032315 | Cyp1a1 |
| ENSMUSG00000039004 | Bmp6 |
| ENSMUSG00000057933 | Gsta2 |
| ENSMUSG00000018924 | Alox15 |
| ENSMUSG00000016756 | Cmah |
| ENSMUSG00000079554 | Aox2 |
| ENSMUSG00000028687 | Mutyh |
| ENSMUSG00000000320 | Alox12 |
| ENSMUSG00000066071 | Cyp4a12a |
| ENSMUSG00000023926 | Rhag |
| ENSMUSG00000027983 | Cyp2u1 |
| ENSMUSG00000026177 | Slc11a1 |
| ENSMUSG00000020891 | Alox8 |
| ENSMUSG00000032807 | Alox12b |
| ENSMUSG00000057880 | Abat |
| ENSMUSG00000025701 | Alox5 |
| ENSMUSG00000032323 | Cyp11a1 |
